# Spin Coupling in Symmetric and Asymmetric Allyl and Phenalenyl Diradicals Bridged by an Inverted Singlet–Triplet System

**DOI:** 10.1021/acs.jpca.6c00123

**Published:** 2026-03-06

**Authors:** Marco Tommaso Barreca, Francesco Di Maiolo

**Affiliations:** Department of Chemistry, Life Science and Environmental Sustainability, 271577 Università di Parma, Parma 43124, Italy

## Abstract

Organic diradicals bridged by inverted singlet–triplet (InveST) units have recently emerged as promising molecular platforms for spin–optical functionality, enabling optical control of spin–spin interactions within a fully organic framework. Here, we study a series of symmetric and asymmetric InveST-bridged diradicals based on the smallest InveST motif, 1,3-diazete, functionalized with allyl and phenalenyl radical units. Within the Pariser–Parr–Pople (PPP) framework, these systems possess disjoint diradical ground states with degenerate singlet and triplet levels, while population of the bridge LUMO triggers finite exchange interactions that stabilize the triplet excited state. Torsional flexibility at the bridge–radical junctions plays a key role in triggering spin–orbit coupling, thereby promoting intersystem crossing between the lowest excited singlet and triplet states. Through evaluation of intersystem crossing and reverse intersystem crossing rates using a vibronic model, we find that all three diradicals support net population transfer from the singlet to the triplet manifold under ambient conditions.

## Graphical abstract

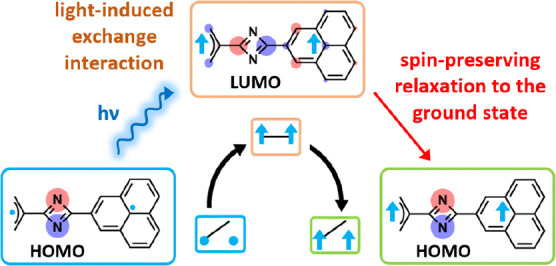

## 1 Introduction

Optically addressable electron spins that can be initialized and detected using light constitute promising candidates for qubits. In such systems, commonly referred to as color centers, spin-dependent optical transitions together with spin-selective intersystem crossing (ISC) enable optical spin initialization, microwave-driven spin manipulation, and spin-state readout via spin-dependent photoluminescence, as realized in optically detected magnetic resonance (ODMR) experiments. Among these platforms, the nitrogen–vacancy center in diamond has emerged as the prototypical example, demonstrating single-spin addressability, long coherence times, and high-fidelity optical readout.^1–4^

Despite their successes, defect-based solid-state systems are inherently constrained in terms of chemical tunability and bottom-up scalability, prompting increasing interest in molecular alternatives. High-spin transition-metal complexes have replicated several key properties of spin–photon interfaces; however, metal-centered platforms frequently exhibit reduced spin coherence times, in addition to concerns related to cost, elemental availability, and long-term sustainability.^5–16^ Metal-free organic diradicals have emerged as a promising molecular realization of color centers.^17–33^ Achieving robust ODMR in these systems remains challenging, requiring three key ingredients: (i) a high-spin ground state, (ii) spin-selective interactions among excited states, and (iii) irreversible relaxation back to the ground state. Organic diradicals naturally satisfy the first requirement; however, achieving a high-spin ground state typically relies on a large spatial separation between the unpaired electrons to suppress direct radical–radical exchange. This separation is often retained in the excited state manifold, inhibiting the spin-selective interactions necessary for ODMR functionality.

To overcome this limit, building on the strategy first introduced in ref 33, we study disjoint organic diradicals in which the radical centers are not directly coupled, but instead connected through a conjugated molecular bridge characterized by an inverted singlet–triplet (InveST) energy gap.^34,35^ In this work, we focus on a bridge derived from the smallest system known to exhibit this property, namely 1,3-diazete (see Figure 1a).^36^ The 1,3-diazete unit displays a pronounced complementarity between its frontier molecular orbitals (MOs), giving rise to a first excited state with multiresonant charge-transfer (MRCT) character, in which electron density is transferred from the HOMO to the LUMO. More generally, this HOMO–LUMO complementarity has been identified as a hallmark of polyenes with alternating electron-donor and electron-acceptor units, with 1,3-diazete representing the minimal molecular motif that exhibits such behavior.^36,37^ The 1,3-diazete ring is intrinsically strained and has been predicted to be thermodynamically destabilized due to angle strain and localized π-electron repulsion.^38–40^ However, substitution and conjugation are known to substantially modulate the energetics of small C_2_N_2_ heterocycles.

**Figure 1:**
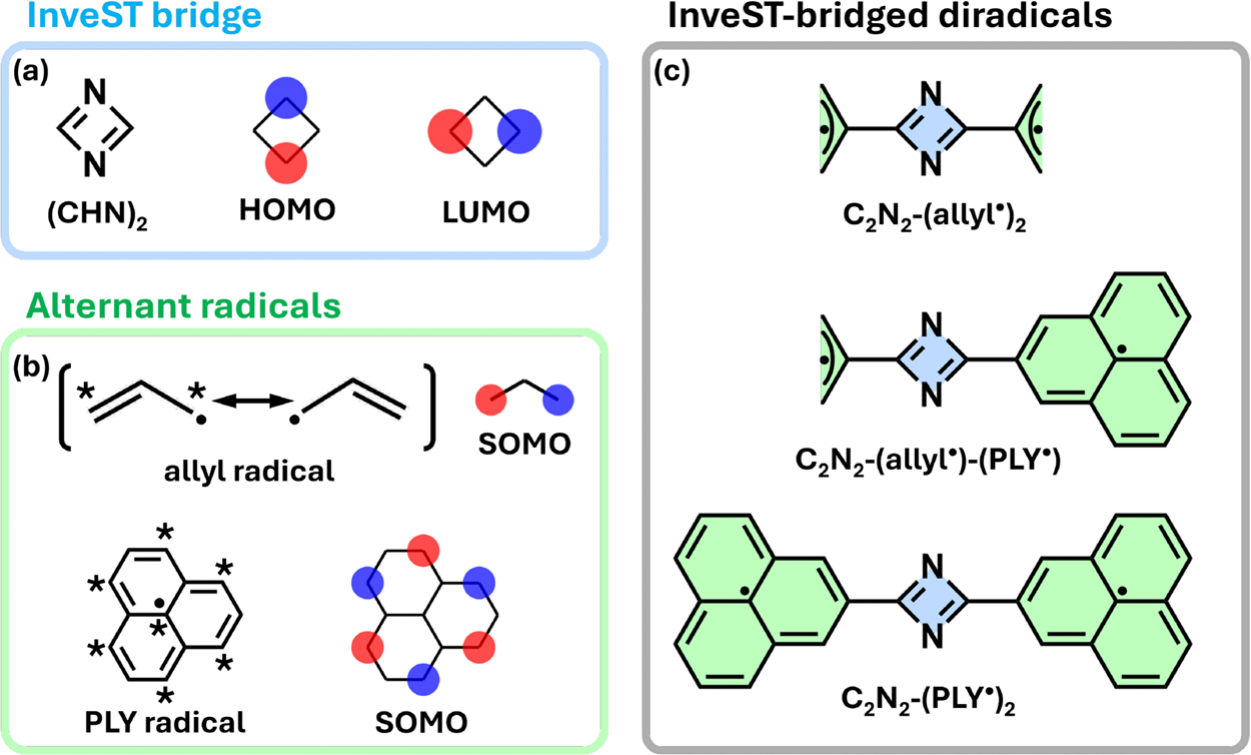
(a) Chemical structure of the minimal InveST system, 1,3-diazete, along with its frontier molecular orbitals obtained from PPP Hartree–Fock calculations. (b) Representative alternant hydrocarbon radicals—allyl and phenalenyl (PLY)—showing their corresponding SOMOs. (c) Molecular structures of the three InveST-bridged diradicals studied in this work.

As radical units, we use alternant hydrocarbon radicals, whose electronic structures are particularly favorable for maintaining spin separation and for enabling well-controlled exchange interactions.^20–22,41–45^ In these systems, the carbon lattice can be partitioned into two complementary sublattices arranged such that each carbon atom is bonded only to atoms belonging to the opposite set (see Figure 1b). This alternant topology results in a nonbonding singly occupied molecular orbital (SOMO) that is confined to a single sublattice, specifically the one containing the greater number of atomic sites.

By connecting the spin centers to sites where the bridge LUMO is localized, spin–spin communication is expected to be active only in the excited state. Consistent with this design, the absence of common overlap regions between the bridge HOMO and the radical-units SOMO is expected to yield nearly degenerate open-shell singlet *S*_0_ and triplet *T*_0_ ground states. Upon excitation, population of the bridge LUMO is expected to activate a finite exchange interaction with the two SOMOs, enabling optical control of the spin–spin coupling via a HOMO→LUMO transition. Because the bridge possesses an inverted singlet–triplet ordering, the lowest HOMO→LUMO excitation localized on the spacer is singlet in character. When this singlet bridge excitation couples via exchange to the two radical spins, the total spin manifold reorganizes such that an overall triplet excited state is stabilized relative to the corresponding singlet configuration. This design therefore enables photoswitchable control of the magnetic coupling between paramagnetic centers.

Spin–optical interfaces support spin-dependent interactions within the excited-state manifold, giving rise to different population and relaxation pathways for different spin configurations and thereby fulfilling the second requirement for ODMR. A key mechanism enabling this behavior is ISC, which is driven by spin–orbit coupling (SOC) and allows communication between singlet and triplet states. Although SOC is formally absent in ideal, planar π-conjugated frameworks, even modest departures from planarity introduce the symmetry breaking required for singlet–triplet mixing. InveST-bridged disjoint diradicals present inherent torsional flexibility about the bonds linking the radical moieties to the central core. At ambient temperature, thermal fluctuations trigger these torsional modes, providing an effective pathway for SOC, enhancing ISC. Across the relevant torsional configurations, both the *S*_1_ and *T*_1_ states retain their diradical nature: the unpaired spins remain localized on separate radical units, with no significant intramolecular SOMO–SOMO charge transfer.

By combining the Pariser–Parr–Pople (PPP) model^46–48^ with high-level multireference ab initio methods, we show that InveST-bridged disjoint diradicals can provide a possible molecular platform for implementing ODMR-relevant spin–optical functionality. The PPP framework is employed as a chemically transparent π-electron model to explore structure–property relationships and torsion-dependent spin–orbit effects, while CASSCF/QD-NEVPT2 calculations are used to benchmark and validate the qualitative electronic-structure picture. Our calculations indicate that SOC–mediated ISC between the *S*_1_ and *T*_1_ states can proceed efficiently, giving rise to excited-state dynamics compatible with ODMR schemes that are able of generating triplet ground-state spin polarization. We discuss this design strategy using 1,3-diazete covalently linked to allyl and phenalenyl (PLY) radical units (see Figure 1c), establishing this molecular architecture as a potential platform for spin-based sensing and information-processing applications.

## 2 Theoretical Methods

The PPP model provides a minimal description of π-conjugated systems with interacting electrons. Like the Hückel approach, it considers only the 2*p*_*z*_ atomic orbitals (AOs) oriented perpendicular to the molecular plane at each atomic site. In contrast to the Hückel model, the PPP framework explicitly includes electron–electron interactions within the zero differential overlap approximation, whereby overlap integrals between 2*p*_*z*_ orbitals on different atoms are neglected. The PPP Hamiltonian in the AO basis reads:

$$H_{PPP}=\underset{\mu }{\sum }\varepsilon_{\mu }n_{\mu }-\underset{\mu \nu ,\nu >\mu }{\sum }\underset{\sigma }{\sum }t_{\mu \nu }\left(a_{\mu \sigma }^{†}a_{\nu \sigma }+a_{\nu \sigma }^{†}a_{\mu \sigma }\right)+\underset{\mu }{\sum }U_{\mu }n_{\mu ↑}n_{\mu ↓}+\underset{\mu \nu ,\nu >\mu }{\sum }V_{\mu \nu }\left(Z_{\mu }-n_{\mu }\right)\left(Z_{\nu }-n_{\nu }\right)$$ 1

where *a*_μσ_^(†)^ annihilates (creates) an electron with spin σ on atomic site μ, and the total electron population at that site is defined as *n*_μ_ = ∑_σ_ *a*_μσ_^†^ *a*_μσ_. The first term of the Hamiltonian contains the on-site orbital energy ε_μ_ associated with the 2*p*_*z*_ orbital on atom μ, together with the hopping integral *t*_μν_ describing electron transfer between neighboring sites μ and ν. Within the PPP formalism, hopping is restricted to atom pairs connected by a σ bond. The second term accounts for electron–electron interactions: *U*_μ_ denotes the on-site Coulomb repulsion between two electrons occupying the same 2*p*_*z*_ orbital, whereas *V*_μν_ represents the intersite Coulomb interaction between electrons localized on atoms μ and ν (see Supporting Information (SI), Section S1.1). The parameter *Z*_μ_ corresponds to the effective nuclear charge at site μ, obtained after subtracting the contribution of the π electrons, with *Z*_μ_ = 1 for both carbon and aza-nitrogen atoms.

We rewrite the PPP Hamiltonian in the MO picture by introducing the operator *b*_*k*σ_^(†)^ = ∑_μ_ *c*_μ,*k*_ *a*_μσ_^(†)^ that annihilates (creates) an electron with spin σ in the *k*-th MO, and where *c*_kμ_ are the expansion coefficients of the *k*-th MO on the AOs obtained upon diagonalization of the relevant Fock operator (see SI, Section S1.1). The resulting Hamiltonian is written on the basis defined by the Hartree–Fock ground state configuration and the single, double, triple, etc. excited configurations obtained by promoting one, two, three, etc. electrons from the occupied to the virtual MOs.^36^

The minimal C_2_N_2_–(allyl^•^)_2_ system allows for an exact (full-CI) treatment within the PPP framework. Direct comparison with full-CI results shows that the CISDTQ expansion is effectively converged for this system, reproducing the full-CI electronic structure at a reduced computational cost (see SI, Section S1.2). Accordingly, PPP–CISDTQ is used for studying both ground- and excited-state properties of C_2_N_2_–(allyl^•^)_2_.

For the larger C_2_N_2_–(allyl^•^)–(PLY^•^) and C_2_N_2_–(PLY^•^)_2_ diradicals, where a full CISDTQ treatment becomes computationally prohibitive, we instead employ the Restricted Active Space Configuration Interaction (RASCI) approach in the hole–particle approximation.^37,49^ Within this framework, the PPP MOs are partitioned into three subspaces of increasing energy – RAS1, RAS2, and RAS3. RAS1 comprises the lowest-energy occupied orbitals, RAS2 includes the highest occupied and lowest virtual orbitals, and RAS3 contains the remaining high-energy virtual orbitals. All possible electronic configurations within the RAS2 subspace are explicitly included, ensuring a complete treatment of the active orbitals most relevant to the low-energy electronic structure. Additional correlation effects are incorporated through the hole–particle approximation, which allows for configurations involving holes in RAS1 and electrons promoted into RAS3 (see SI, Section S1.3). Comparison with multireference ab initio CASSCF/QD-NEVPT2 calculations is carried out for all systems to assess the accuracy of the PPP-based descriptions. In addition to the minimal CASSCF­(4,4) active space employed for the main analysis, selected calculations were performed using an enlarged CASSCF­(6,6) space to verify the robustness of the results with respect to active space size (see SI Section S2.2).

## 3 Results

### 3.1 The PPP Model Parametrization

Modeling organic diradicals bridged by InveST motifs poses a significant challenge for quantum chemical approaches, owing to their intrinsically multiconfigurational electronic structure and strong electron–electron correlations.^33^ As a first step, we focus on the simplest system shown in Figure 1c, namely C_2_N_2_–(allyl^•^)_2_. Its limited size—ten sites and ten π electrons—allows for an exact treatment (i.e., full-CI) within the PPP framework. Direct comparison with the full-CI results shows that the CISDTQ expansion is effectively converged for this system, reproducing the full-CI electronic structure while reducing the problem size (see SI, Section S1.2). Accordingly, PPP–CISDTQ is used for the analysis of both ground- and excited-state properties of C_2_N_2_–(allyl^•^)_2_.

The parameters associated with carbon atoms are well established in the literature and are known to be highly transferable.^50–52^ Accordingly, the carbon site energy ε_C_ is taken as the reference zero, the on-site Coulomb repulsion is set to *U*_C_ = 11.26 eV, and the nearest-neighbor C–C hopping integral is fixed at *t* = −2.4 eV. The same hopping value is adopted for C–N bonds. To remain consistent with standard PPP parametrizations and to isolate the electronic effects of π conjugation, all molecular geometries are idealized by setting bond angles to 120° and bond lengths to 1.4 Å. A quantitative comparison with ab initio DFT-optimized geometries is provided in Section S1.1 of the Supporting Information. A more subtle aspect concerns the on-site energy of the nitrogen atoms, for which PPP parametrization is considerably less standardized and no universally accepted values exist.^51–63^ To address this point, we carried out an exploratory study of the (*U*_N_, ε_N_) parameter space for C_2_N_2_–(allyl^•^)_2_ by monitoring the dependence of the energy gaps Δ_*S*_0_–*T*_0__, Δ_*S*_1_–*T*_1__, Δ_*T*_1_–*T*_0__, and Δ_*S*_1_–*S*_0__ on the nitrogen site energy ε_N_ and the on-site Coulomb repulsion *U*_N_, as shown in Figure 2.

**Figure 2:**
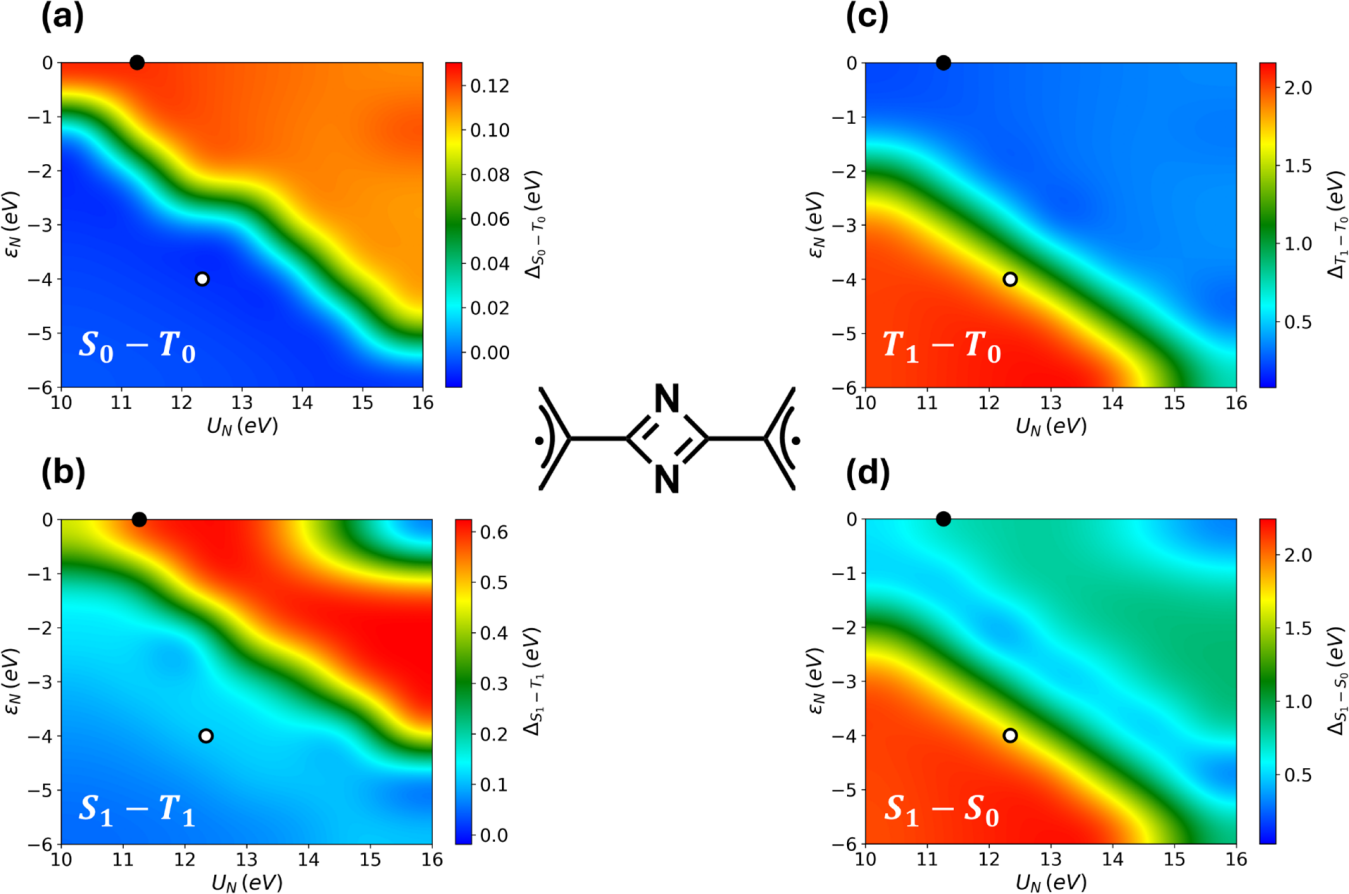
Exploratory study for C_2_N_2_-(allyl^•^)_2_. Color maps illustrate how (a) the Δ_ST_ gap in the ground state, (b) the excited state Δ_ST_ gap, (c) the transition energy between *T*_0_ and *T*_1_, and (d) the transition energy between *S*_0_ and *S*_1_ vary with *U*_N_ and ε_N_. Calculation performed at the PPP-CISDTQ level, using the model parameters specified in the main text.

To quantify the diradical character of the system and evaluate the reliability of the model, Figure 2a reports the behavior of the ground state singlet–triplet gap Δ_*S*_0_–*T*_0__ as a function of ε_N_ and *U*_N_. The black marker indicates the parameter set corresponding to carbon atoms (ε_N_ = 0 and *U*_N_ = 11.26 eV), for which Δ_*S*_0_–*T*_0__ is positive and amounts to 0.123 eV, indicating a triplet ground state. As ε_N_ becomes increasingly negative and *U*_N_ is reduced—corresponding to progressively more electron-withdrawing atoms at the bridge nitrogen positions—the gap decreases and approaches zero. The white marker denotes a physically realistic parameter set for ε_N_ and *U*_N_ reported for porphines in ref 62. In particular, the on-site Coulomb repulsion is taken directly from that work (*U*_N_ = 12.34 eV), while the site energy is slightly shifted from ε_N_ = −3.2 eV to ε_N_ = −4 eV to achieve better agreement with ab initio calculations. For this parameter set, the *S*_0_ and *T*_0_ states are nearly degenerate (Δ_*S*_0_–*T*_0__ = 0.009 eV), consistent with a disjoint diradical ground state. A comparison with multireference ab initio calculations at the CASSCF/QD-NEVPT2 level yields a closely comparable gap of 0.021 eV. In this parameter range, communication between the two spin centers is effectively suppressed in the ground state. By contrast, the corresponding excited-state energy gap Δ_*S*_1_–*T*_1__, shown by the white dot in panel b, amounts to 0.121 eV, demonstrating that a substantial spin–spin exchange interaction is activated upon excitation. The corresponding CASSCF/QD-NEVPT2 value of 0.19 eV confirms the stabilization of the *T*_1_ excited state with respect to *S*_1_.

The physical origin of this excitation-induced spin–spin interaction can be understood by examining the nature of the low-lying electronic excitations at the white marker position. Here, the lowest energy excitation is predominantly localized on the InveST bridge and corresponds to a HOMO→LUMO transition. Population of the bridge LUMO in the excited state enables exchange interactions with the radicals SOMOs, thereby activating spin communication that is absent in the ground state. CI weights (see SI, Section S4) show that both the *S*_1_ and *T*_1_ states are mainly described by the HOMO→LUMO excitation. Crucially, these excited states retain a diradical character: the two unpaired electrons remain spatially localized on distinct radical units rather than forming an intramolecular SOMO→SOMO charge-transfer configuration. An explicit search for an inter-SOMO charge-transfer singlet state shows that this configuration lies significantly higher in energy than the HOMO→LUMO *S*_1_/*T*_1_ pair (see SI, Section S5). This picture also explains the trends observed in the excitation energies reported in panels c and d. Starting from the black marker (carbon-like parameter set), the *S*_0_–*S*_1_ and *T*_0_–*T*_1_ energy gaps are relatively small, with the triplet excitation lying significantly lower in energy (Δ_*T*_1_–*T*_0__ = 0.26 eV and Δ_*S*_1_–*S*_0__ = 0.75 eV). As the nitrogen site energy ε_N_ becomes more negative, stabilization of the bridge HOMO leads to a systematic increase in both excitation energies. Upon reaching the white marker, the *T*_0_–*T*_1_ and *S*_0_–*S*_1_ gaps in panels c and d amount to 1.564 and 1.694 eV, respectively, in qualitative agreement with the corresponding multireference ab initio results (see Section 3.2).

### 3.2 Torsional Modulation of Electronic Energies and SOC

A key requirement for realizing spin–optical functionality in molecular systems is the presence of spin-selective ISC between singlet and triplet manifolds, a process governed by SOC. In InveST-bridged diradicals, SOC does not arise in the planar geometry but is instead triggered by thermally accessible torsional distortions around the bonds connecting the InveST core to the radical units. These out-of-plane fluctuations locally break the planarity of the π-conjugated framework, thereby activating SOC. Owing to the strong 1/*r* ^3^ dependence of SOC on interatomic distance, the dominant contributions are highly localized at the bridge–radical junctions, where structural flexibility is greatest. Within the PPP framework, SOC is introduced explicitly through an additional spin-flipping term in the electronic Hamiltonian and is written as^20,64–67^

$$H_{\mathrm{SOC}}=A\underset{\mu \nu }{\sum }\left(a_{\mu ↑}^{†}a_{\nu ↓}-a_{\nu ↑}^{†}a_{\mu ↓}\right)$$ 2

where the sum runs over neighboring atoms. The SOC matrix element is purely imaginary and depends explicitly on the torsional distortion of the molecular framework through the prefactor *A* = −*i*(3.94 × 10^–4^)­sinθ (in eV), with θ denoting the dihedral angle between the InveST core and the radical units (see Figure 3a). This expression ensures that SOC vanishes for planar geometries and becomes finite only in the presence of out-of-plane torsional motion.

**Figure 3:**
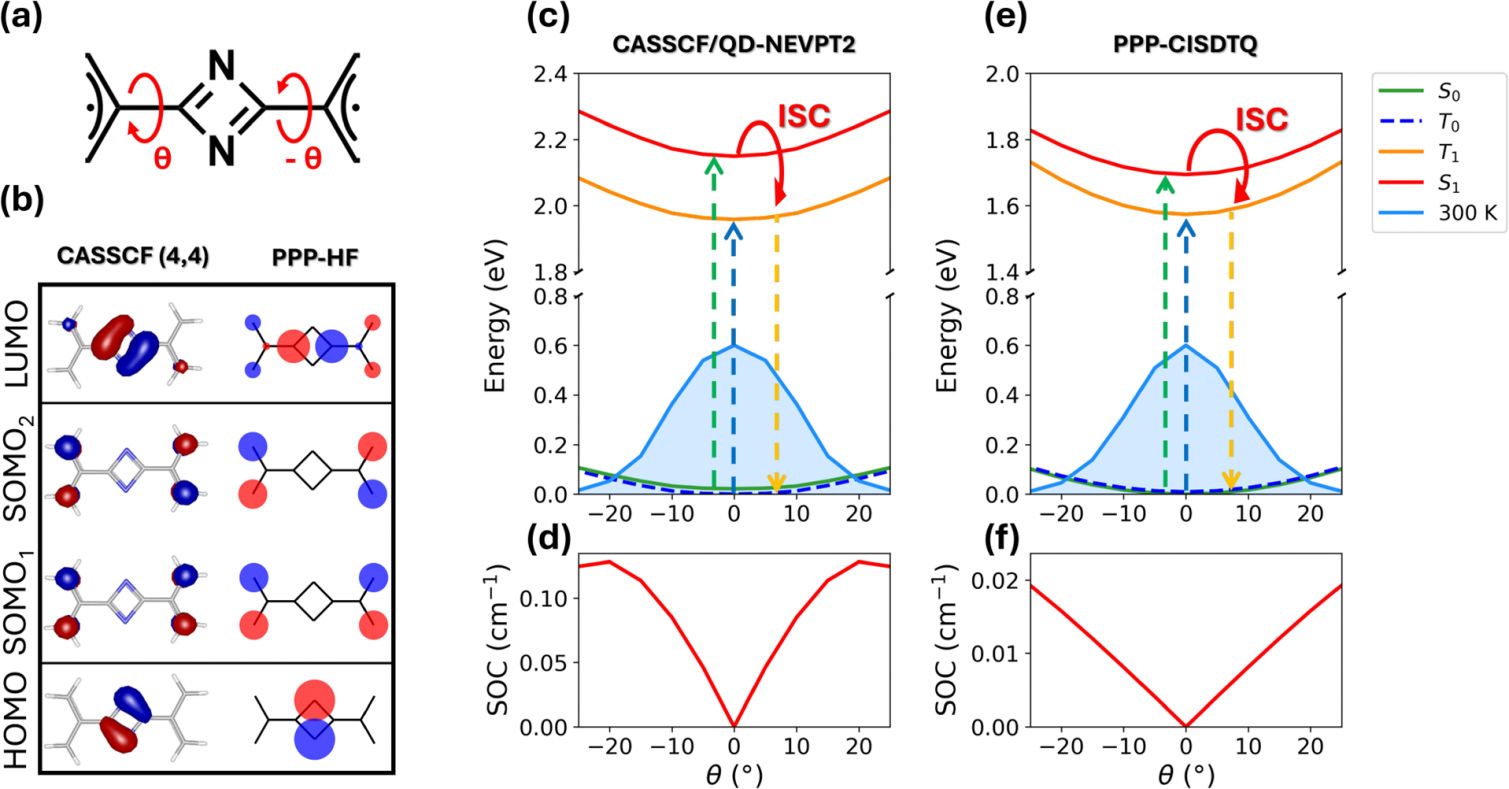
Electronic structure and photophysical properties of the C_2_N_2_-(allyl^•^)_2_ system. (a) Molecular structure highlighting the torsional coordinate θ around the bridge-radical unit bond. (b) Comparison between frontier molecular orbitals, for θ = 0°, obtained at the PPP-HF and CASSCF­(4,4) levels. (c) CASSCF­(4,4)/QD-NEVPT2 potential energy curves for *S*_0_, *T*_0_, *S*_1_, and *T*_1_ and Boltzmann distribution for the ground state at room temperature. (d) SOC behavior, at the same level of theory as above, as a function of the torsional coordinate θ. (e, f) PPP-CISDTQ results corresponding to panels (c, d). Model parameters defined in the main text.

Properly accounting for torsional flexibility is therefore essential for describing SOC-driven spin mixing in these systems. While the PPP model was originally formulated for planar π-conjugated hydrocarbons,^46,47,68^ its extension to nonplanar structures can be achieved by introducing torsional corrections to the electronic hopping integrals. Specifically, the hopping amplitude between adjacent sites is modulated according to the dihedral angle as *t*_μν_(θ) = *t*_μν_ cosθ. In the present model, both InveST–radical dihedral angles are varied by equal magnitudes, irrespective of whether the rotations occur in the same or opposite directions. This prescription ensures that electronic coupling is maximal for fully planar conformations, corresponding to θ = 0° or 180°, and progressively reduced as the molecule twists out of plane. Torsional strain is incorporated through a steric potential of the form *V*_steric_(θ) = sin^2^θ, which is assumed to be identical in both the ground and excited electronic states. Within this description, torsional motion simultaneously modulates the electronic coupling through the cosθ dependence of the hopping integrals and activates SOC via the sinθ dependence of the spin–orbit coupling matrix elements.

In Figure 3, we study the torsional flexibility of C_2_N_2_–(allyl^•^)_2_ (panel a). At the planar geometry, the electronic structure is characterized by frontier orbitals in which both the HOMO and LUMO are mainly localized on the InveST bridge, as shown in panel (b) at both the PPP and CASSCF levels. The two allyl radical units contribute a pair of degenerate singly occupied molecular orbitals, giving rise to their in-phase (SOMO_1_) and out-of-phase (SOMO_2_) combinations. This MO pattern is preserved throughout the entire torsional range explored in this work. Potential energy curves calculated as a function of θ at both the PPP and CASSCF/QD-NEVPT2 levels reveal that the ground and low-lying excited states adopt a planar equilibrium geometry, with θ_eq_ = 0°. Across the entire torsional range, the *S*_0_ and *T*_0_ states remain degenerate, consistent with the absence of spin–spin exchange interactions in the ground state. By contrast, the excited-state manifold exhibits different behavior. Population of the bridge LUMO activates exchange interactions between the two radical centers, enabling spin–spin communication and opening an energy gap between *T*_1_ and *S*_1_. As shown in panels (c) and (e), the triplet excited state *T*_1_ is stabilized relative to *S*_1_ over the full thermally accessible torsional range, with the singlet–triplet splitting Δ_*S*_1_–*T*_1__ reaching its maximum value at the planar geometry.

The torsional dependence of SOC provides further insight into the spin dynamics of this system. The absolute value of the SOC matrix element between the *S*_1_ state and the *M*_*S*_ = ±1 sublevels of the *T*_1_ state is reported in panel (d) at the CASSCF/QD-NEVPT2 level and in panel (f) at the PPP level. In both approaches, no coupling is observed between *S*_1_ and the *M*_*S*_ = 0 component of *T*_1_, in agreement with spin-selection rules.^66^ As expected, the SOC vanishes exactly at θ = 0°, where the system is fully planar. However, even small deviations from planarity immediately generate finite SOC values, indicating that modest torsional fluctuations are sufficient to activate ISC. The quantitative consequences of these couplings for ISC rates are discussed in Section 3.3. At the PPP–CISDTQ level, the SOC magnitude increases monotonically with torsional angle, reaching values of 0.019 cm^–1^ at θ = ±25°. These magnitudes are comparable to SOC strengths reported for thermally activated delayed fluorescence (TADF) molecules known to undergo efficient ISC,^69,70^ supporting the idea that thermally accessible twisting at the InveST–radical junctions can provide an effective pathway for spin-state interconversion. At the ab initio CASSCF/QD-NEVPT2 level, the SOC exhibits a nonmonotonic dependence on θ, reaching a maximum value of 0.128 cm^–1^ around θ ≃ ±20° before decreasing at larger torsional distortions. The differences between the two results reflect the approximate nature of the PPP MOs entering the spin-flipping hopping term, which only partially capture the detailed torsional modulation of SOC in nonplanar geometries. In particular, the zero differential overlap (ZDO) approximation inherent to the PPP model limits the quantitative accuracy of the SOC matrix elements. In the present system, the dominant SOC contributions originate from spin-flip excitations localized within each SOMO, namely SOMO_1_^α^ → SOMO_1_^β^ and SOMO_2_^α^ → SOMO_2_^β^. The overall SOC magnitude therefore reflects the combined contributions of these two localized channels, whose relative phase and amplitude are modulated by the torsional angle at the bridge–radical connections. At the CASSCF/QD-NEVPT2 level, the maximum SOC around θ ≃ 20° corresponds to constructive interference between these contributions, while at larger torsional angles their partial destructive interference leads to a reduction of the coupling.

The electronic structure of the C_2_N_2_–(allyl^•^)_2_ system is well suited to support an ODMR mechanism. In this molecule, *S*_1_ and *T*_1_ are optically dark owing to the symmetric character of the C_2_N_2_ bridge. Consequently, optical excitation proceeds via higher-lying excited states, which subsequently relax to the *S*_1_ and *T*_1_ manifolds through internal conversion (see SI, Section S5). SOC between *S*_1_ and *T*_1_ then enables ISC from *S*_1_ to selected *T*_1_ sublevels (*M*_*S*_ = ±1). This process is followed by nonradiative internal conversion from *T*_1_ to *T*_0_ (dashed orange arrows in panels c and e). Because this relaxation is spin-preserving, the nonthermal population of *T*_1_ sublevels generated by ISC is transferred to the *T*_0_ manifold, resulting in triplet ground state spin polarization. Alternatively, the spin-polarized *T*_1_ manifold could be interrogated directly via photoinduced-absorption-detected magnetic resonance (PADMR) spectroscopy.^71^ Indeed, the metastable *T*_1_ population generated by ISC enables excited-state absorption toward higher-lying triplet states (*T*_1_ → *T*_*n*_), offering an optical handle on the *T*_1_ spin-polarized triplet manifold without relying on light emission.

Having established the reliability of the PPP description for the minimal InveST-bridged diradical, we now turn to larger systems, for which full PPP–CISDTQ calculations become computationally prohibitive. To this end, we employ the PPP–RASCI­(h,p,hp) approach. Using a RAS2 active space comprising four electrons distributed over five orbitals (see SI, Section S1.3), PPP–RASCI is benchmarked against PPP–CISDTQ for the minimal C_2_N_2_–(allyl^•^)_2_ system. In this case, the *S*_0_–*T*_0_ gap changes from 0.009 eV at the PPP–CISDTQ level to 0.005 eV within PPP–RASCI, while the *S*_1_–*T*_1_ gap varies from 0.12 to 0.14 eV, with the electronic character of the relevant states (*S*_0_, *T*_0_, *S*_1_, and *T*_1_) fully preserved. In addition to its accuracy, PPP–RASCI provides a substantial reduction in computational cost. For the minimal C_2_N_2_–(allyl^•^)_2_ system, the number of basis functions in the *S*_*z*_ = 0 sector decreases from 42,588 at the PPP–CISDTQ level to 2700 within the PPP–RASCI­(h,p,hp) framework. This favorable scaling makes PPP–RASCI particularly well suited for extending the present analysis to larger InveST-bridged diradicals.

Both C_2_N_2_–(allyl^•^)–(PLY^•^) (Figure 4a) and C_2_N_2_–(PLY^•^)_2_ (Figure 4c) present electronic structures closely analogous to that of C_2_N_2_–(allyl^•^)_2_. In particular, *S*_0_ and *T*_0_ remain nearly degenerate over the entire torsional range explored, consistent with the disjoint nature of the diradicals and the absence of spin–spin coupling in the ground state. In contrast, an energy gap opens between the lowest excited singlet and triplet states, with *T*_1_ lying below *S*_1_, indicating activation of exchange interactions upon excitation. For the asymmetric C_2_N_2_–(allyl^•^)–(PLY^•^) system, the planar geometry (θ = 0°) yields a degenerate ground state, with Δ_*S*_0_–*T*_0__ = 0.003 eV at the PPP–RASCI level and 0.009 eV at the CASSCF/QD-NEVPT2 level. The corresponding excited-state gap amounts to Δ_*S*_1_–*T*_1__ = 0.105 eV at both theoretical levels (black curve in panel b). Upon introducing thermally accessible torsional distortions, the *S*_1_–*T*_1_ gap is slightly reduced, reaching 0.086 eV at θ = 25°. At the same time, torsional motion activates SOC between *S*_1_ and the *M*_*S*_ = ±1 sublevels of *T*_1_, with the SOC magnitude increasing to approximately 0.009 cm^–1^ at θ = 25° (red curve in panel b). This value is smaller than that obtained at the ab initio CASSCF/QD-NEVPT2 level—where the SOC reaches a maximum of 0.099 cm^–1^ at θ = 20° (see SI, Section S3)—and it reflects the approximate nature of the PPP MOs entering the SOC Hamiltonian.

**Figure 4:**
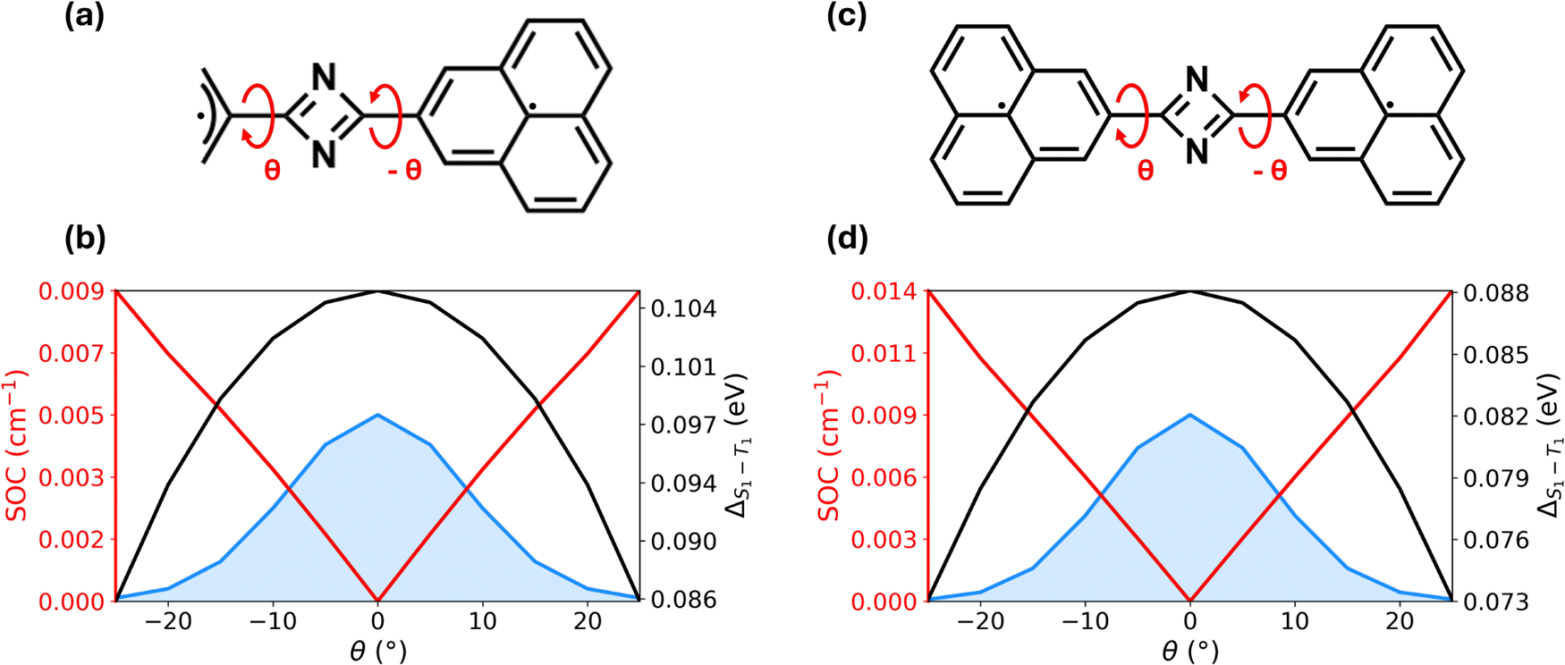
Effect of torsional flexibility in PLY-based InveST-bridged diradicals. (a) Molecular structure of C_2_N_2_–(allyl^•^)–(PLY^•^), highlighting the torsional coordinate θ around the bridge–radical bond. (b) Dependence of the *S*_1_–*T*_1_ energy gap (black curve) and the SOC magnitude (red curve) on the torsional angle θ, together with the ground-state Boltzmann distribution at room temperature (blue shaded area). (c, d) Corresponding results for C_2_N_2_–(PLY^•^)_2_. All calculations are performed at the PPP–RASCI­(h,p,hp) level using the same PPP model parameters as in Figure 3.

A similar behavior is observed for the symmetric C_2_N_2_–(PLY^•^)_2_ system. Here again, the *S*_0_ and *T*_0_ states remain degenerate across the entire torsional range. At the planar geometry, the *S*_1_–*T*_1_ gap amounts to 0.088 eV at the PPP–RASCI level and 0.090 eV at the CASSCF/QD-NEVPT2 level. As the molecule twists out of plane, the excited-state gap decreases, reaching 0.073 eV at θ = 25° (black curve in panel d), while SOC between *S*_1_ and *T*_1_ is progressively activated (red curve in panel d). At θ = 25°, the SOC magnitude reaches approximately 0.014 cm^–1^, which is of the same order of magnitude as the corresponding ab initio value. Indeed, CASSCF/QD-NEVPT2 calculations predict a maximum SOC of 0.046 cm^–1^ at θ = 20° (see SI, Section S3).

Overall, these results demonstrate that the key features identified for the minimal C_2_N_2_–(allyl^•^)_2_ system—namely, ground-state spin decoupling, excited-state exchange activation, and torsion-induced SOC—are preserved upon increasing the size and asymmetry of the radical units.

For completeness, a modified attachment topology was also examined in which the PLY radical units are connected to the InveST bridge through sites with nonzero SOMO amplitude (see SI, Section S6). In this case, strong mixing between radical and bridge MOs leads to loss of the disjoint diradical character and a closed-shell singlet ground state.

Building on our previous work,^33^ where the SOMO–LUMO exchange integral was shown to provide a reliable descriptor of excited-state spin–spin interaction strengths in InveST-bridged diradicals, we apply the same analysis to the three systems discussed here (see Figure 5). Specifically, we calculate the SOMO–LUMO exchange integral at the PPP level (see SI, Section S1.4) as a function of the torsional coordinate for C_2_N_2_–(allyl^•^)_2_, C_2_N_2_–(allyl^•^)–(PLY^•^), and C_2_N_2_–(PLY^•^)_2_ (panel a). Across the entire torsional range explored, the exchange integral follows the trend C_2_N_2_–(allyl^•^)_2_ > C_2_N_2_–(allyl^•^)–(PLY^•^) > C_2_N_2_–(PLY^•^)_2_, highlighting the direct correlation between the magnitude of the exchange interaction and the extent of SOMO–LUMO spatial overlap (panel b). Among the three systems, C_2_N_2_–(allyl^•^)_2_ exhibits the largest SOMO–LUMO overlap, resulting in the strongest excited-state coupling between the radical centers. In contrast, the more extended C_2_N_2_–(PLY^•^)_2_ framework displays the weakest orbital overlap and, consequently, the smallest exchange interaction. The asymmetric C_2_N_2_–(allyl^•^)–(PLY^•^) system shows intermediate behavior, remaining closer to the allyl-only case due to the presence of the allyl radical unit, which enhances orbital overlap with the bridge LUMO. To further validate this result, we calculated the exchange integral using CASSCF­(4,4) MOs and the Multiwfn package.^72,73^ The resulting values (panels c and d) confirm the same qualitative trend and span a comparable energy range.

**Figure 5:**
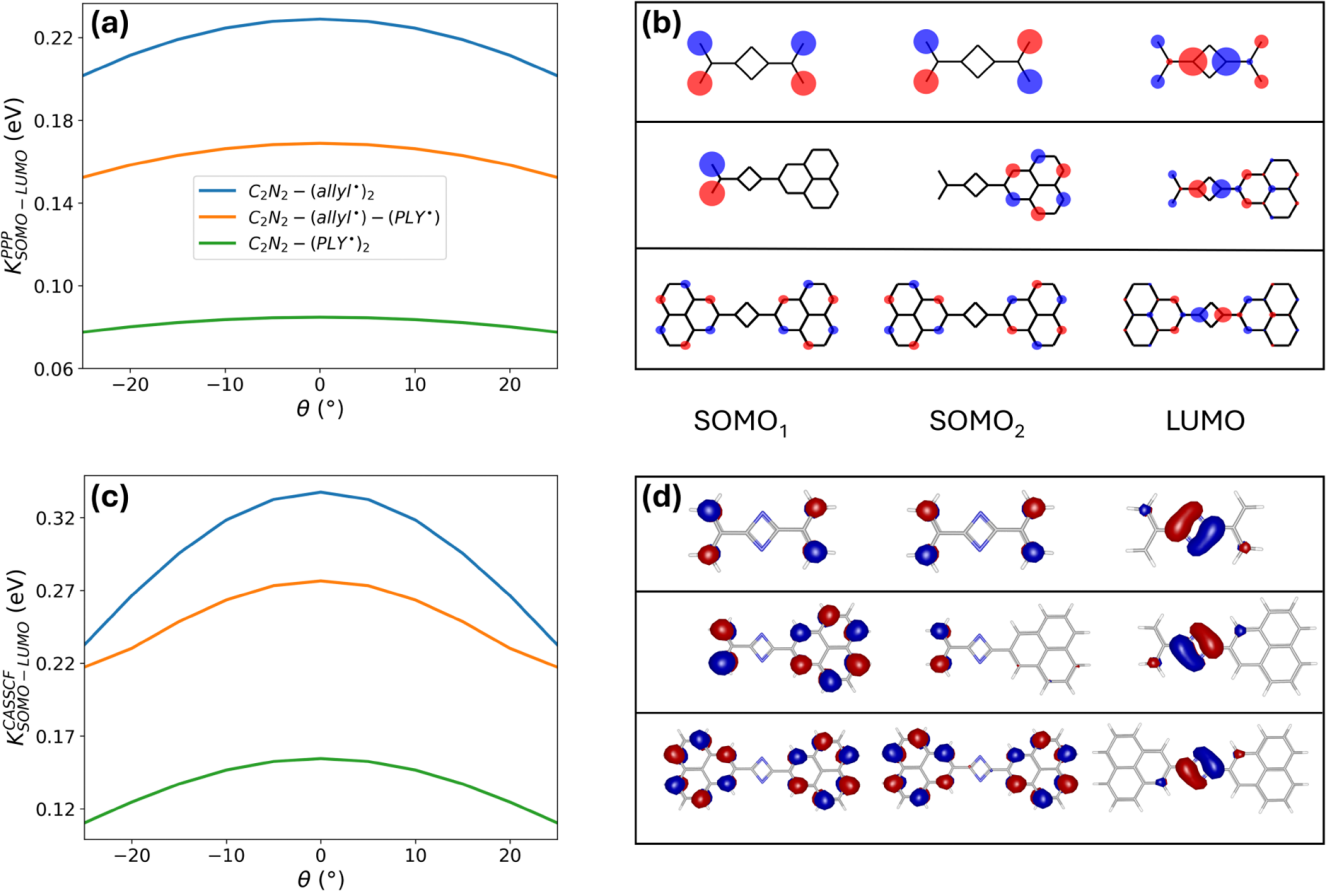
Exchange interactions in C_2_N_2_-bridged diradicals. (a) SOMO-LUMO exchange integral as a function of torsional angle θ for C_2_N_2_-(allyl^•^)_2_ (blue), C_2_N_2_-(allyl^•^)-(PLY^•^) (orange), and C_2_N_2_-(PLY^•^)_2_ (green) at the PPP Hartree–Fock level. (b) PPP frontier MO sketches (SOMO_1_, SOMO_2_, LUMO) for the three systems at θ = 0°. (c, d) Relevant results obtained with CASSCF MOs (active space (4,4)). Same PPP model parameters used in Figure 3.

### 3.3 ISC Dynamics and Spin Polarization Mechanism

To study the spin-dependent processes underlying ODMR mechanism, we calculate the ISC and reverse ISC (RISC) rates for the three C_2_N_2_-bridged diradicals. In these systems, spin polarization emerges from the combined effect of torsional flexibility, excited-state exchange interactions, and SOC, all of which are thermally modulated at ambient conditions. The degenerate *S*_0_/*T*_0_ ground-state manifold spans a wide range of torsional conformations at the bridge–radical junctions, which in turn modulate both the *S*_1_–*T*_1_ energy gap and the SOC magnitude.

For all three diradicals, the lowest singlet and triplet excited states are optically dark (see SI, Section S5), such that photoexcitation proceeds via higher-lying states, followed by rapid, spin-preserving internal conversion into the *S*_1_ and *T*_1_ manifolds. Once populated, these states form the central hub of the spin polarization mechanism. Torsional motion activates SOC, enabling ISC from *S*_1_ to the *M*_*S*_ = ±1 sublevels of *T*_1_, while the *S*_1_–*T*_1_ energy gap controls the competition between ISC and RISC.

Finally, the spin polarization established in the excited state must be transferred back to the ground-state manifold without compromising its spin-selective character. In InveST-bridged diradicals, this condition is inherently satisfied because SOC between *S*_0_ and *T*_0_ vanishes as a consequence of the nodal structure of the HOMO and SOMOs, preventing spin mixing during decay. Below, we quantify these processes by evaluating ISC and RISC rates for the three systems discussed above.

We begin by diabatizing the potential energy surfaces associated with *S*_0_, *T*_0_, *S*_1_, and *T*_1_. The resulting model consists of four diabatic states: two singlets—a neutral configuration |^1^ *N*⟩ and a multiresonant charge-transfer configuration |^1^MRCT⟩—and their triplet counterparts, |^3^ *N*⟩ and |^3^MRCT⟩. The neutral diabatic states |^1^ *N*⟩ and |^3^ *N*⟩ are taken as the zero of energy, while the diabatic energies of the charge-transfer states are set to 2*z* for |^1^MRCT⟩ and 2*s* for |^3^MRCT⟩. Coupling between the singlet diabatic states is described by a θ-dependent matrix element −τ­(θ), whereas the corresponding coupling within the triplet manifold is given by −β­(θ). SOC is introduced in accordance with El-Sayed’s rule by coupling |^1^ *N*⟩ with |^3^MRCT⟩ and |^3^ *N*⟩ with |^1^MRCT⟩ through a constant matrix element *V*_SOC_. The value of *V*_SOC_ is determined by requiring that the SOC matrix element |⟨*S*_1_|*V*_SOC_|*T*_1_⟩|, evaluated from the eigenstates of the diabatic Hamiltonian, reproduces the torsional dependence of the SOC obtained from the PPP–RASCI calculations. The diabatic Hamiltonian is expressed in the basis {|^1^ *N*⟩, |^3^ *N*⟩, |^1^MRCT⟩, |^3^MRCT⟩} and reads:

$$H=\left(\begin{array}{cccc} 0 & 0 & -\tau (\theta ) & V_{\mathrm{SOC}}\\ 0 & 0 & V_{\mathrm{SOC}} & -\beta (\theta )\\ -\tau (\theta ) & V_{\mathrm{SOC}} & 2z & 0\\ V_{\mathrm{SOC}} & -\beta (\theta ) & 0 & 2s \end{array}\right)+\frac{\hbar \omega_{t}}{2}\left(\theta^{2}+p_{\theta }^{2}\right)+a\theta^{4}$$ 3

where the torsional coordinate θ is treated as a vibrational degree of freedom with frequency ω_*t*_, and *p*_θ_ denotes its conjugate momentum. The torsional modulation of the electronic couplings is described by −τ­(θ) = −τ_0_ cos­(2θ)­sin­(2θ) for the singlet manifold and by −β­(θ) = −β_0_ cos­(2θ)­sin­(2θ) for the triplet manifold. To reproduce the shape of the ground- and excited-state potential energy profiles along θ, an anharmonic quartic restoring potential is employed. Additional details are reported in the SI, Section S7.1.

ISC and RISC processes are mediated by relatively weak SOC and can therefore be treated within a perturbative framework. To evaluate the corresponding transition rates, we first diagonalize the Hamiltonian in eq 3 with the SOC term *V*_SOC_ set to zero. Under this condition, the singlet and triplet subspaces are fully decoupled and can be studied independently. The resulting vibronic eigenstates belonging to the *S*_1_ and *T*_1_ manifolds are shown as black lines in Figure 6a,c,e.

**Figure 6:**
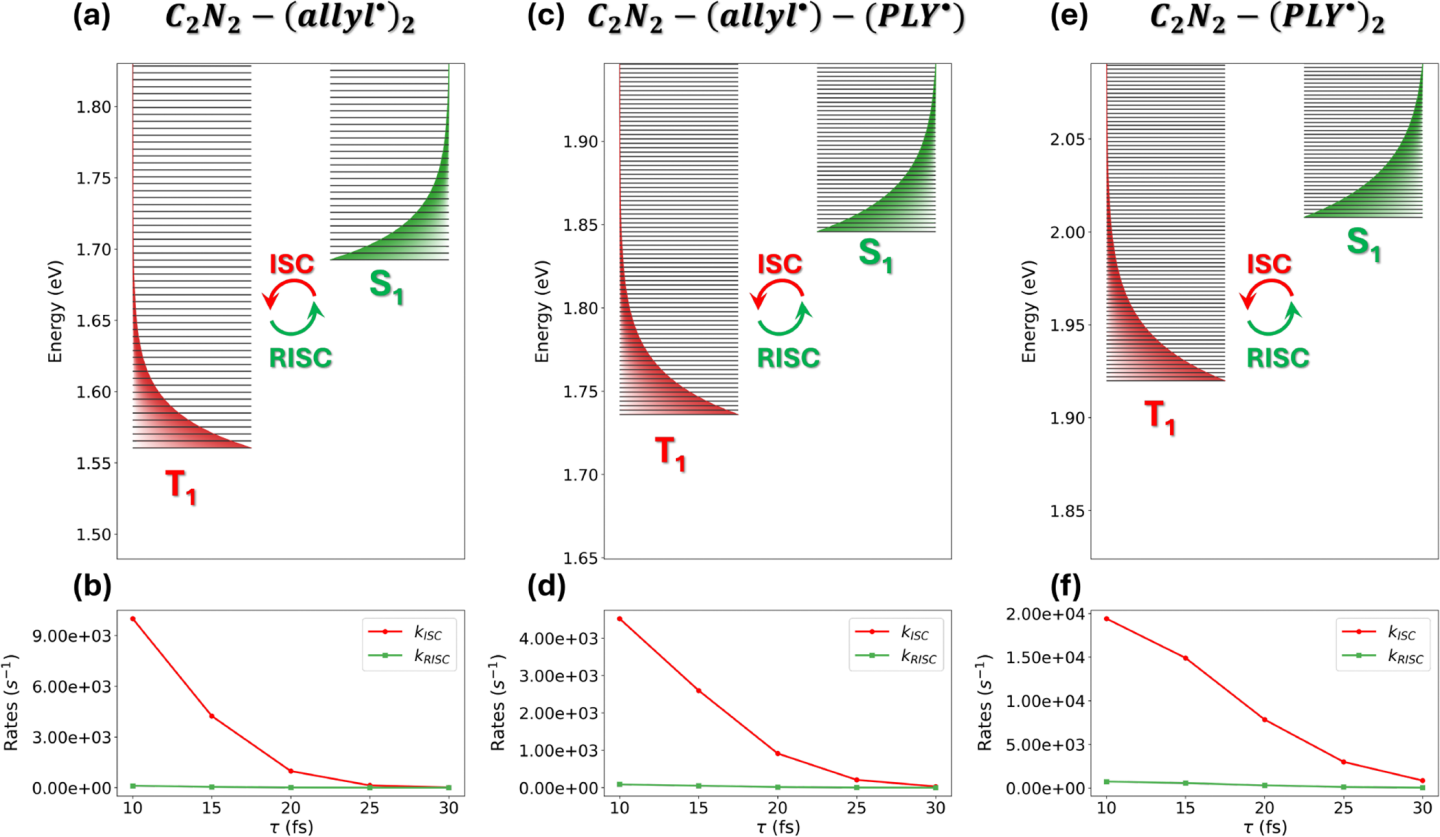
Vibronic framework used to calculate ISC and RISC rates for C_2_N_2_–(allyl^•^)_2_ (panel a), C_2_N_2_–(allyl^•^)–(PLY^•^) (panel c), and C_2_N_2_–(PLY^•^)_2_ (panel e), based on the PPP electronic structure results discussed above. Red and green curves denote the energies of the vibronic triplet (*T*_1_) and singlet (*S*_1_) eigenstates, respectively. The total ISC rate is obtained by summing all *S*_1_ → *T*_1_ transition rates and averaging over the Boltzmann distribution of the singlet states, represented schematically by the green shaded region. RISC rates are derived from the corresponding ISC rates by enforcing the condition of microscopic reversibility. ISC and RISC rates computed for different values of the relaxation time τ are shown in panels b, d, and f. Parameters for C_2_N_2_-(allyl^•^)_2_: τ_0_ = 0.34 eV, β_0_ = 0.44 eV, 2*z* = 1.69 eV, 2*s* = 1.58 eV, ℏω_*t*_ = 7.31 × 10^–4^ eV, *a* = −0.21 eV, *V*_SOC_ = −0.08 eV. Parameters for C_2_N_2_-(allyl^•^)-(PLY^•^): τ_0_ = 0.50 eV, β_0_ = 0.56 eV, 2*z* = 1.85 eV, 2*s* = 1.75 eV, ℏω_*t*_ = 3.64 × 10^–4^ eV, *a* = 0.68 eV, *V*_SOC_ = −0.03 eV. C_2_N_2_-(PLY^•^)_2_: τ_0_ = 0.46 eV, β_0_ = 0.51 eV, 2*z* = 2.00 eV, 2*s* = 1.93 eV, ℏω_*t*_ = 3.01 × 10^–4^ eV, *a* = −0.04 eV, *V*_SOC_ = −0.06 eV.

Because internal conversion within a given spin manifold occurs on ultrafast time scales—typically tens of femtoseconds— we assume that ISC originates from a thermally equilibrated population of *S*_1_ vibronic states. This equilibrium distribution is illustrated by the green shaded regions in Figure 6a,c,e. Individual singlet-to-triplet transition rates are then calculated using the Fermi Golden Rule *k*_ISC_^*i*→*j*^ = |⟨*i*|*V*_SOC_|*j*⟩|^2^ *S*_*ij*_2π/ℏ where *S*_*ij*_ denotes the overlap between the vibronic states |*i*⟩ and |*j*⟩. Each vibronic level is represented by a Gaussian line shape with width σ, related to the relaxation time τ through $\sigma ={\left(2\pi \tau \sqrt{2\ln2}\right)}^{-1}$ . RISC rates are obtained from the corresponding ISC rates by enforcing detailed balance. The resulting ISC and RISC rates, evaluated for five representative values of τ, are reported in Figure 6b,d,f. The chosen range of relaxation times (10–30 fs) is consistent with realistic vibronic lifetimes in organic molecules.^74,75^

For all three diradicals analyzed at the PPP level, the calculated ISC and RISC rates decrease systematically with increasing relaxation time τ and eventually converge toward zero. In particular, at τ = 30 fs both processes yield vanishing and nearly identical rate constants. This behavior reflects the combined effect of the progressively narrower Gaussian lineshapes associated with longer relaxation times (i.e., smaller σ) and the sizable *S*_1_–*T*_1_ energy separation, which together suppress the overlap between vibronic levels and strongly reduce the efficiency of both ISC and RISC.

At shorter relaxation times, a markedly different regime emerges. For smaller values of τ, the ISC rate increases substantially, while the corresponding RISC rate remains negligible at room temperature. For example, at τ = 10 fs the symmetric C_2_N_2_–(allyl^•^)_2_ system exhibits an ISC rate on the order of 10^4^ s^–1^, whereas the RISC rate remains effectively zero. In the asymmetric C_2_N_2_–(allyl^•^)–(PLY^•^) diradical, the reduced SOC magnitude obtained at the PPP level leads to a lower ISC efficiency, with rates reaching only ∼5 × 10^3^ s^–1^ at τ = 10 fs. By contrast, for C_2_N_2_–(PLY^•^)_2_, the smaller *S*_1_–*T*_1_ energy gap partially compensates for the reduced SOC, yielding an ISC rate of approximately 2 × 10^4^ s^–1^ at the same relaxation time. To further assess the role of temperature, the ISC and RISC rates were evaluated in the 100–300 K range at fixed τ = 10 fs. The ISC rates decrease moderately with lowering temperature but remain within the same order of magnitude, whereas the RISC rates remain negligible throughout this interval (see SI, Section S7.3).

ISC and RISC rates computed using ab initio QD-NEVPT2 input data are reported in the SI (Section S7.2) and display the same qualitative trends, albeit with systematically larger ISC values. At τ = 10 fs, the ISC rate increases from 6 × 10^4^ s^–1^ for C_2_N_2_–(allyl^•^)_2_ to 4 × 10^5^ s^–1^ for C_2_N_2_–(allyl^•^)–(PLY^•^), and remains sizable at 2 × 10^5^ s^–1^ for C_2_N_2_–(PLY^•^)_2_. In contrast, RISC rates remain negligible at ambient temperature across the entire range of relaxation times considered, indicating a robust bias toward net population transfer from the singlet to the triplet manifold.

## 4 Conclusions

In this work, we have studied a family of InveST-bridged organic diradicals featuring allyl and phenalenyl radical units as molecular platforms for spin–optical functionality relevant to ODMR mechanisms. By extending our previous design strategy to symmetric and asymmetric diradicals based on the smallest InveST bridge, 1,3-diazete, we have shown that controlled spin–spin interactions and spin-selective excited-state dynamics can be achieved within a fully organic framework.

Using a combination of the PPP model and multireference ab initio calculations, we showed that all three systems—C_2_N_2_–(allyl^•^)_2_, C_2_N_2_–(allyl^•^)–(PLY^•^), and C_2_N_2_–(PLY^•^)_2_—exhibit disjoint diradical ground states with nearly degenerate *S*_0_ and *T*_0_ levels, resulting from the absence of orbital overlap between the bridge HOMO and the radical SOMOs. In contrast, population of the bridge LUMO in the excited state activates finite exchange interactions, stabilizing the *T*_1_ state relative to *S*_1_ and enabling optical control of spin–spin coupling. The magnitude of this interaction follows a clear trend that correlates with the extent of SOMO–LUMO overlap, as quantified through the SOMO–LUMO exchange integral.

Torsional flexibility at the bridge–radical junctions was shown to play a central role in enabling SOC, thereby activating ISC between the *S*_1_ state and the *M*_*S*_ = ±1 sublevels of *T*_1_. Our analysis demonstrates that even modest, thermally accessible deviations from planarity are sufficient to induce SOC while preserving the diradical character of the excited states. The resulting SOC strengths and *S*_1_–*T*_1_ energy gaps place these systems in a regime favorable to an efficient ISC without significant RISC at ambient temperature. By calculating ISC and RISC rates within a vibronic framework, we established that all three diradicals support net population transfer from the singlet to the triplet manifold, a key prerequisite for ground-state spin polarization.

Overall, our results show that InveST-bridged diradicals based on the minimal 1,3-diazete core, combined with chemically tunable radical units, constitute a versatile and scalable molecular platform for achieving spin-selective optical control in purely organic systems. These findings further expand the design space for molecular spin–optical interfaces and highlight the potential of InveST-based architectures for future applications in molecular quantum sensing and spin-based information processing.

In particular, the presence of a excited triplet manifold that can be selectively spin-polarized at room temperature through SOC-mediated ISC suggests the potential for magnetic-field-sensitive spectroscopy in fully organic systems, without relying on defect-based solid-state platforms. The ability to prepare a nonthermal distribution of triplet sublevels under ambient conditions provides a foundation for microwave manipulation of optically initialized spin states. Furthermore, the subsequent transfer of this polarization to the ground state triplet manifold through spin-preserving internal conversion offers a route toward optical initialization of a multilevel spin system,^76^ which may serve as a starting point for qudit-like encoding schemes and for the development of room-temperature sensing protocols based on purely organic architectures. Practical realization of these architectures would most naturally involve solid-state, crystalline, or matrix environments, where environmental confinement may further enhance the structural robustness of the substituted C_2_N_2_ bridge. We hope that the electronic design principles established here will stimulate experimental and synthetic efforts toward the development of stable InveST-based spin-optical interfaces.

## Supplementary Material



## Data Availability

The data that support the findings of this study are available from the corresponding author upon reasonable request.

## Notes

Technical details on the PPP model implementation (Section S1); additional CASSCF/QD-NEVPT2 results obtained using different active spaces (Section S2); supplementary torsional scan results for the C_2_N_2_–(allyl^•^)–(PLY^•^) and C_2_N_2_–(PLY^•^)_2_ diradicals (Section S3); the electronic character of the *S*_1_ and *T*_1_ excited states (Section S4); higher-lying excited states (Section S5); results for a system in which the phenalenyl radical units are attached to the InveST bridge through atomic sites bearing finite SOMO amplitude (Section S6); the calculation of ISC and RISC rate constants (Section S7); and the Cartesian coordinates and calculated vibrational frequencies of the DFT-optimized geometries (Section S8) (PDF)
