# Supplementary material for: Spin Coupling in Symmetric and Asymmetric Allyl and Phenalenyl Diradicals Bridged by an Inverted Singlet–Triplet System
Source: J Phys Chem A. 2026 Mar 6;130(11):2399–410. doi: 10.1021/acs.jpca.6c00123 (PMC13007031; doi:10.1021/acs.jpca.6c00123)
Supplement: Supplementary file 1 [file jp6c00123_si_001.pdf]

Supplementary Information

Spin Coupling in Symmetric and Asymmetric Allyl and Phenalenyl Diradicals Bridged by an Inverted Singlet–Triplet System

Marco Tommaso Barreca and Francesco Di Maiolo*

Department of Chemistry, Life Science and Environmental Sustainability, Università di Parma, 43124 Parma, Italy.

E-mail: francesco.dimaiolo@unipr.it

Table of Contents

S1 Details of the PPP Model Implementation **S3**

S1.1 The PPP-CI formulation S3

S1.2 The PPP–CI Expansion for C_2N_2–(allyl$^\bullet$)$_2$ S7

S1.3 The PPP-RASCI approach S9

S1.4 The PPP SOMO-LUMO exchange integral S13

S2 *Ab initio* Computational Details **S14**

S2.1 Computational Setup and Electronic Structure Methods S14

S2.2 Active Space Dependence: CASSCF Molecular Orbitals and QD-NEVPT2 Energies S15

S3 Additional Torsional Scan Results **S20**

S4 Wavefunction Analysis of the S_1 and T_1 Excited States **S22**

S5 Higher-lying excited states **S24**

S6 Effect of Radical Attachment Topology **S27**

S7 ISC and RISC Rate Calculation Details **S29**

S7.1 Construction of the Diabatic Hamiltonian S29

S7.2 Vibronic Model for Torsional Dynamics S29

S7.3 Temperature dependence of ISC and RISC rates S31

S8 Cartesian Coordinates and Vibrational Frequencies **S33**

References **S56**

S1 Details of the PPP Model Implementation

S1.1 The PPP-CI formulation

In the real-space formulation of the PPP Hamiltonian (Eq. 1 in the main text), the inter-site Coulomb interaction $V_{\mu\nu}$ is described using the Ohno parametrization.[1–3] Within this scheme, the electron–electron repulsion between sites μ and ν separated by a distance $r_{\mu\nu}$ is given by:

$$V_{\mu\nu} = \frac{e^2}{4\pi\varepsilon_0} \left[r_{\mu\nu}^2 + \left(\frac{\varepsilon_r e^2}{4\pi\varepsilon_0 (U_\mu + U_\nu)} \right)^2 \right]^{-1/2} \tag{S1}$$

where the relative dielectric constant is set to $\varepsilon_r = 2$ to represent screening effects typical of organic molecular environments.[3]

Because the Ohno parametrization explicitly depends on the interatomic distances $r_{\mu\nu}$, a consistent molecular geometry must be specified for the PPP simulations. In line with standard PPP practice and in order to isolate purely electronic effects associated with π conjugation, all structures were described using an idealized geometry in which C–C and C–N bond lengths are fixed to 1.4Å. Bond angles are set to 90° within the central C_2N_2 four-membered ring and to 120° elsewhere, consistent with the local hybridization pattern.

Table S1: Deviations between DFT-optimized heavy-atom geometries and the idealized PPP reference (hydrogens omitted). PPP bond lengths are fixed to 1.4Å. Bond angles are fixed to 90° within the central C_2N_2 four-membered ring and to 120° elsewhere.

System	Bond lengths vs PPP		Bond angles vs PPP	
	RMSD (Å)	Max $\|\Delta r\|$ (Å)	RMSD (°)	Max $\|\Delta\alpha\|$ (°)
C_2N_2-(allyl$^\bullet$)$_2$	0.06097	0.10340	7.759	12.726
C_2N_2-(allyl$^\bullet$)-(PLY$^\bullet$)	0.04163	0.10507	4.988	12.977
C_2N_2-(PLY$^\bullet$)$_2$	0.03376	0.10391	3.951	12.692

A quantitative comparison between these idealized geometries and DFT-optimized heavy-atom structures is provided in Table S1. Across all three diradicals, deviations in bond lengths are modest, with root-mean-square differences below 0.07Å and maximum deviations of approximately 0.10Å. Bond angle deviations are likewise limited, with RMSD values of

only a few degrees and maxima below 13°. These results indicate that the PPP reference geometries provide a reasonable structural representation of the three molecules. In all cases, the equilibrium structure remains planar, with a torsional angle $\theta = 0°$ at the bridge–radical junctions, consistent with the energy minima discussed in the main text.

To check the impact of the geometric deviations, additional PPP-RASCI(h,p,hp) calculations were performed using the DFT-optimized geometries for all three diradicals. The resulting absolute energies are reported in Table S2. In all cases, the qualitative electronic structure remains unchanged with respect to the idealized geometry. The ground-state S_0 and T_0 states remain nearly degenerate, with splittings of 5 meV, 2 meV, and 1 meV for C_2N_2–(allyl$^\bullet$)$_2$, C_2N_2–(allyl$^\bullet$)–(PLY$^\bullet$), and C_2N_2–(PLY$^\bullet$)$_2$, respectively. Likewise, a finite S_1-T_1 gap is preserved, with values of 140 meV, 95 meV, and 66 meV for the three systems.

Table S2: Absolute energies (in eV) of the lowest singlet and triplet states (S_0, T_0, S_1, and T_1) of C_2N_2-(allyl$^\bullet$)$_2$, C_2N_2-(allyl$^\bullet$)-(PLY$^\bullet$), and C_2N_2-(PLY$^\bullet$)$_2$, computed at the PPP-RASCI(h,p,hp) level using the DFT-optimized geometries in Tables S10, S13, and S16.

	**C_2N_2-(allyl$^\bullet$)$_2$**	**C_2N_2-(allyl$^\bullet$)-(PLY$^\bullet$)**	**C_2N_2-(PLY$^\bullet$)$_2$**
$E(S_0)$	-28.061325	-53.513334	-78.670276
$E(T_0)$	-28.056463	-53.510937	-78.668839
$E(T_1)$	-26.328899	-51.565139	-76.627121
$E(S_1)$	-26.188754	-51.469824	-76.561571

The PPP Hamiltonian is constructed in an atomic orbital (AO) representation that explicitly includes all many-electron configurations generated by distributing n π electrons over N atomic sites. The many-body basis is organized according to eigenstates of the spin projection operator S_z. Spin multiplicities are identified by diagonalizing the Hamiltonian separately within fixed-S_z subspaces: singlet states appear only in the $S_z = 0$ sector, whereas triplet states are present in $S_z = 0$ and $S_z = \pm 1$ sectors. Because the dimension of the Hilbert space increases exponentially with system size, the resulting Hamiltonian matrices become extremely large, albeit highly sparse. Low-lying eigenvalues and eigenvectors are therefore calculated using the implicitly restarted Lanczos algorithm as implemented in the ARPACK library.[4]

To facilitate direct comparison with standard electronic-structure approaches and to maintain computational feasibility for larger systems, the Hamiltonian is recast in a molecular orbital (MO) representation. Within the Hartree–Fock approximation, the PPP Hamiltonian of Eq. 1 (main text) reduces to an effective one-electron Fock operator:

$$F_{PPP} = \sum_{\mu} \left(\varepsilon_{\mu} + J_{\mu\mu} - K_{\mu\mu}\right) n_{\mu} + \sum_{\mu\nu,\mu\neq\nu} \left(-t_{\mu\nu} - K_{\mu\nu}\right) \sum_{\sigma} \left(a^{\dagger}_{\mu\sigma} a_{\nu\sigma} + a^{\dagger}_{\nu\sigma} a_{\mu\sigma}\right) \tag{S2}$$

In accordance with the PPP formalism, the zero-differential overlap approximation is adopted, such that the Coulomb operator becomes diagonal:

$$J_{\mu\mu} = \sum_{\lambda=1}^{N} \left(P_{\lambda\lambda} - Z_{\lambda}\right) V_{\lambda\mu} \tag{S3}$$

and the exchange operator becomes:

$$K_{\mu\nu} = \left(P_{\mu\nu}/2 - Z_{\nu}\delta_{\mu\nu}\right) V_{\mu\nu} \tag{S4}$$

The density matrix elements appearing in Eqs. S3 and S4 are defined as:

$$P_{\mu\nu} = 2 \sum_{k=1}^{SOMO-2} c_{k\mu} c_{k\nu} + c_{SOMO_1,\mu} c_{SOMO_1,\nu} + c_{SOMO_2,\mu} c_{SOMO_2,\nu} \tag{S5}$$

In the first term, the index k runs over the fully occupied MOs of the ground-state configuration, whereas the remaining two terms account for the doubly degenerate SOMOs associated with the two radical units. Self-consistency of the Fock operator is obtained through a variational optimization procedure.

Diagonalization of the Fock operator yields the PPP Hartree–Fock MOs associated with the reference ground-state configuration $|g\rangle$. On this basis, a many-electron Hilbert space is constructed by systematically generating configurations obtained through excitations from occupied to virtual MOs, including single, double, triple, and higher-order excitations. To

this end, we introduce fermionic annihilation (creation) operators $b_{k\sigma}^{(\dagger)}$, which act on the k-th MO and are defined as linear combinations of the AO operators $a_{\mu\sigma}^{(\dagger)}$.

The PPP Hamiltonian in the MO basis reads:

$$
\begin{aligned}
H_{PPP}^{CI} = & \sum_{ij}\sum_{\sigma}\left(\sum_{\mu}\varepsilon_{\mu}c_{\mu,i}c_{\mu,j}\right)b_{i\sigma}^{\dagger}b_{j\sigma} \\
& -\sum_{ij}\sum_{\sigma}\left[\sum_{\mu\nu,\mu\neq\nu}t_{\mu\nu}(c_{\mu,i}c_{\nu,j}+c_{\nu,i}c_{\mu,j}\right]b_{i\sigma}^{\dagger}b_{j\sigma} \\
& +\sum_{ijkl}(\sum_{\mu}U_{\mu}c_{\mu,i}c_{\mu,j}c_{\mu,k}c_{\mu,l})b_{i\uparrow}^{\dagger}b_{j\uparrow}b_{k\downarrow}^{\dagger}b_{l\downarrow} \\
& +\sum_{ijkl}\sum_{\sigma\sigma'}\left(\sum_{\mu,\nu,\mu\neq\nu}\frac{V_{\mu\nu}}{2}c_{\mu,i}c_{\mu,j}c_{\nu,k}c_{\nu,l}\right)b_{i\sigma}^{\dagger}b_{j\sigma}b_{k\sigma'}^{\dagger}b_{l\sigma'} \\
& -\sum_{ij}\sum_{\sigma}\left[\sum_{\mu,\nu,\mu\neq\nu}\frac{V_{\mu\nu}}{2}(Z_{\nu}c_{\mu,i}c_{\mu,j}+Z_{\mu}c_{\nu,i}c_{\nu,j})\right]b_{i\sigma}^{\dagger}b_{j\sigma}
\end{aligned}
\tag{S6}
$$

The PPP Hamiltonian is then diagonalized within a Configuration Interaction (CI) framework to obtain the correlated ground state and the lowest-lying excited states.

S1.2 The PPP–CI Expansion for C_2N_2–(allyl$^\bullet$)$_2$

Here, we discuss the convergence of the PPP excited state energies with respect to the truncation level of the CI expansion for the minimal C_2N_2–(allyl$^\bullet$)$_2$ diradical. Owing to its reduced size – ten π electrons distributed over ten sites – this system allows for an exact solution of the PPP Hamiltonian at the full-CI level, which serves as a rigorous reference.

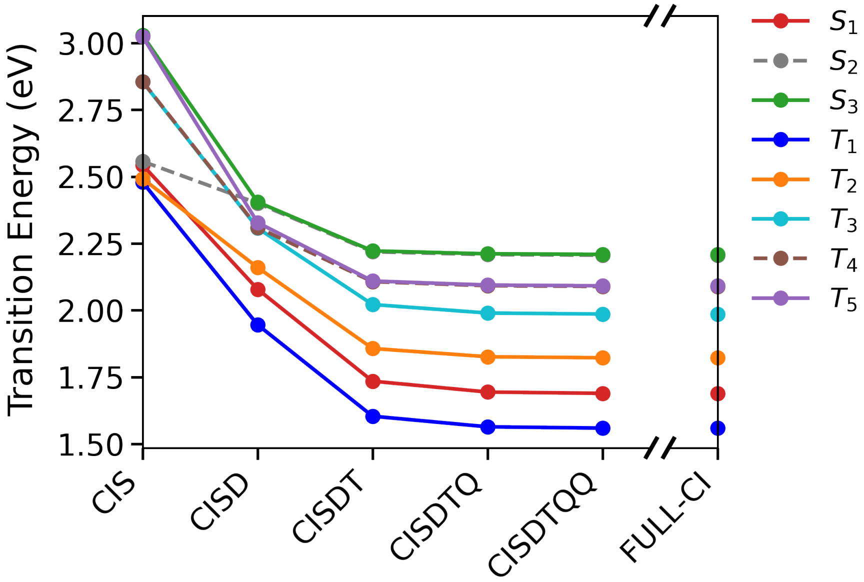

Figure S1: Excitation energies of the first few electronic states of C_2N_2–(allyl$^\bullet$)$_2$ at various theoretical levels. PPP model parameters are specified in the main text.

Figure S1 shows the evolution of the lowest singlet and triplet excitation energies as the PPP-CI expansion is systematically extended from single excitations (CIS) to include double (CISD), triple (CISDT), quadruple (CISDTQ), and quintuple (CISDTQQ) excitations, alongside the corresponding full-CI results. At the CIS level, the excited state ordering is qualitatively incorrect with respect to full-CI. Inclusion of double excitations restores the correct ordering of the low-lying singlet and triplet states. Quantitative convergence of the excitation energies, however, requires the inclusion of higher-order excitations. The transition energies approach the full-CI limit upon inclusion of triple excitations and become quantitatively converged when quadruple excitations are accounted for. Accordingly, PPP-CISDTQ provides an optimal compromise between accuracy and computational efficiency for

C_2N_2–(allyl$^\bullet$)$_2$. In the largest spin sector ($S_z = 0$), the CISDTQ expansion involves 42,588 determinants, compared to 63,504 determinants in the corresponding full-CI space, resulting in a reduction of the computational cost without loss of accuracy. For this reason, all PPP results for C_2N_2–(allyl$^\bullet$)$_2$ reported in the main text are obtained at the PPP–CISDTQ level.

S1.3 The PPP-RASCI approach

To reduce the computational cost of PPP-based calculations while retaining an accurate description of electron correlation, we adopt the Restricted Active Space Configuration Interaction (RASCI) approach within the PPP framework, following the implementation introduced in Ref. 5. In this formulation, the Hartree–Fock MOs obtained from the PPP Hamiltonian are partitioned into three energy-ordered subsets: RAS1, RAS2, and RAS3. The RAS1 subspace contains the lowest-energy occupied orbitals, RAS2 includes a selected set of frontier orbitals comprising both occupied and low-lying virtual MOs, and RAS3 is formed by the remaining higher-energy virtual orbitals.

As in any other single-reference method belonging to the CI family, the RASCI wave function is generated by applying an excitation operator R to the reference configuration. This operator is naturally expanded according to the number of electrons promoted from occupied to virtual orbitals, thereby introducing hole–particle excitations of increasing order within the chosen active space:[6–8]

$$R = r_0 + r_h + r_p + r_{hp} + r_{2h} + r_{2p} + r_{2hp} + \dots \tag{S7}$$

where the leading term, r_0, accounts for all possible excitations confined entirely within the RAS2 subspace. This term therefore spans a reduced full configuration-interaction expansion equivalent to a complete active space CI (CASCI), as it introduces no holes in RAS1 and no particles in RAS3. The remaining contributions progressively incorporate configurations involving holes in the RAS1 space and electrons promoted into the RAS3 space, with the subscripts indicating the number and type of such hole and particle excitations included.

Including higher-order excitation classes systematically improves the level of electron correlation described by the RASCI wave function. As an illustration, the RASCI(h,p) approximation permits at most a single hole in RAS1 or a single particle in RAS3, whereas the RASCI(h,p,hp) variant further allows simultaneous single-hole and single-particle excitations

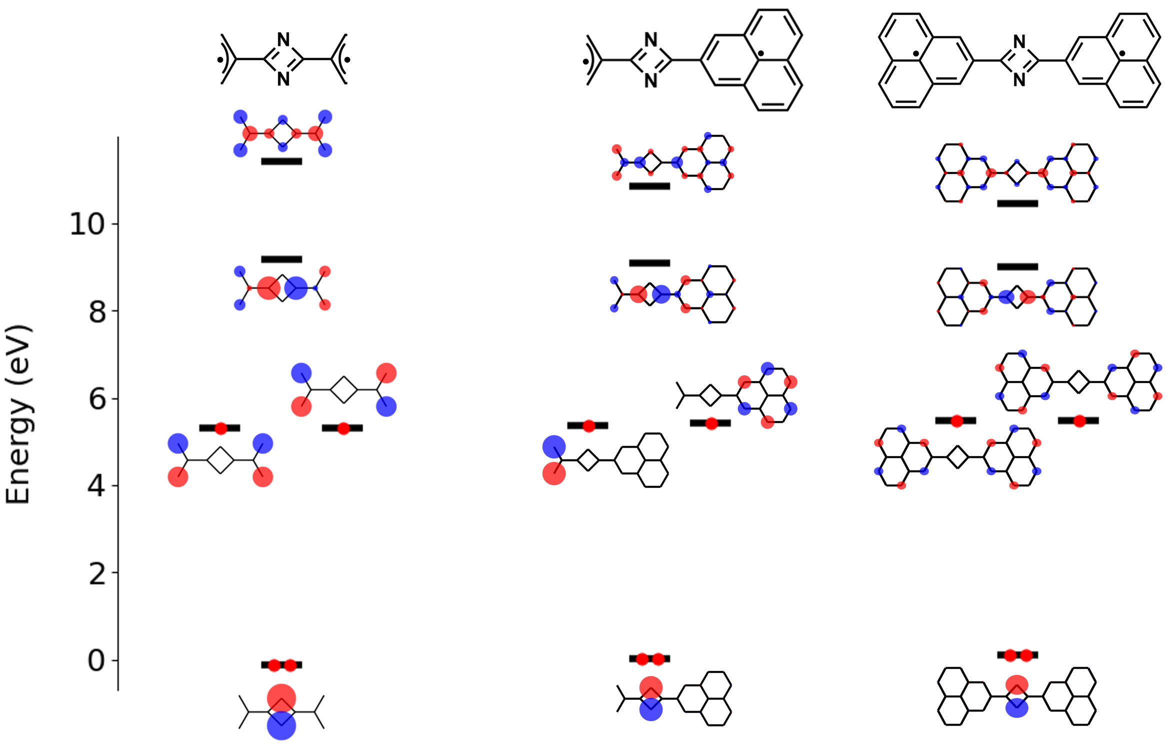

Figure S2: PPP–Hartree–Fock frontier MOs forming the RAS2 subspace for C_2N_2–(allyl$^\bullet$)$_2$, C_2N_2–(allyl$^\bullet$)–(PLY$^\bullet$), and C_2N_2–(PLY$^\bullet$)$_2$ are displayed together with their associated orbital energies. Red markers denote the orbital occupation, with one and two dots indicating singly and doubly occupied orbitals, respectively. All calculations employ the same PPP parameter set as used in the main text.

connecting RAS1 and RAS3. The overall accuracy of the approach is therefore sensitive to both the choice of MOs and the distribution of electrons among the RAS subspaces. In the present work, the active space is defined by a RAS2 sector comprising five MOs occupied by four electrons. With this selection, the PPP–RASCI(h,p,hp) calculations reproduce reference trends obtained from high-level multireference *ab initio* methods. The PPP–Hartree–Fock MOs assigned to the RAS2 subspace are displayed in Fig. S2. For completeness, the full sets of PPP–HF MOs for the three diradicals considered are reported in Figs. S3, S4, and S5, from which the orbitals belonging to the RAS1 and RAS3 subspaces can be readily identified.

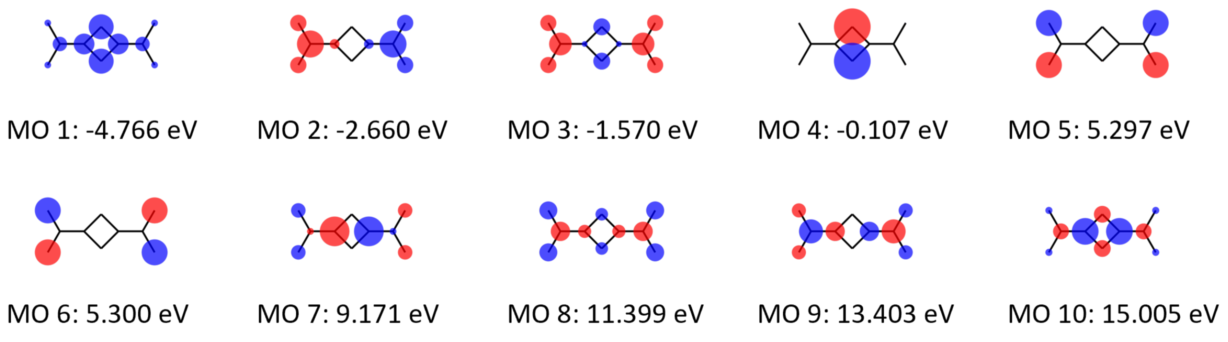

Figure S3: Molecular orbitals obtained at the PPP–Hartree–Fock level for C_2N_2–(allyl$^\bullet$)$_2$, shown together with their corresponding orbital energies. Orbital 4 corresponds to the HOMO, orbitals 5 and 6 are the degenerate SOMO$_1$ and SOMO$_2$, and orbital 7 is the LUMO. The PPP model parameters are the same as those used in the main text.

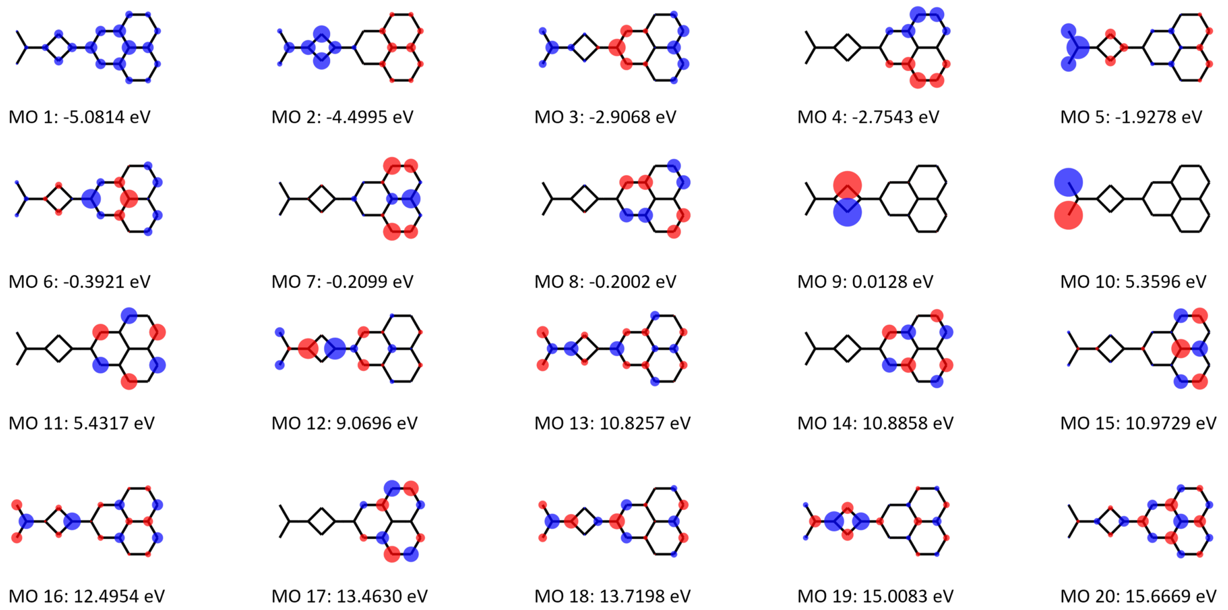

Figure S4: Molecular orbitals obtained at the PPP–Hartree–Fock level for C_2N_2-(allyl$^\bullet$)-(PLY$^\bullet$), shown together with their corresponding orbital energies. MO 9 is the HOMO, MO 10 is SOMO$_1$, MO 11 is SOMO$_2$, and MO 12 is the LUMO. The PPP model parameters are the same as those used in the main text.

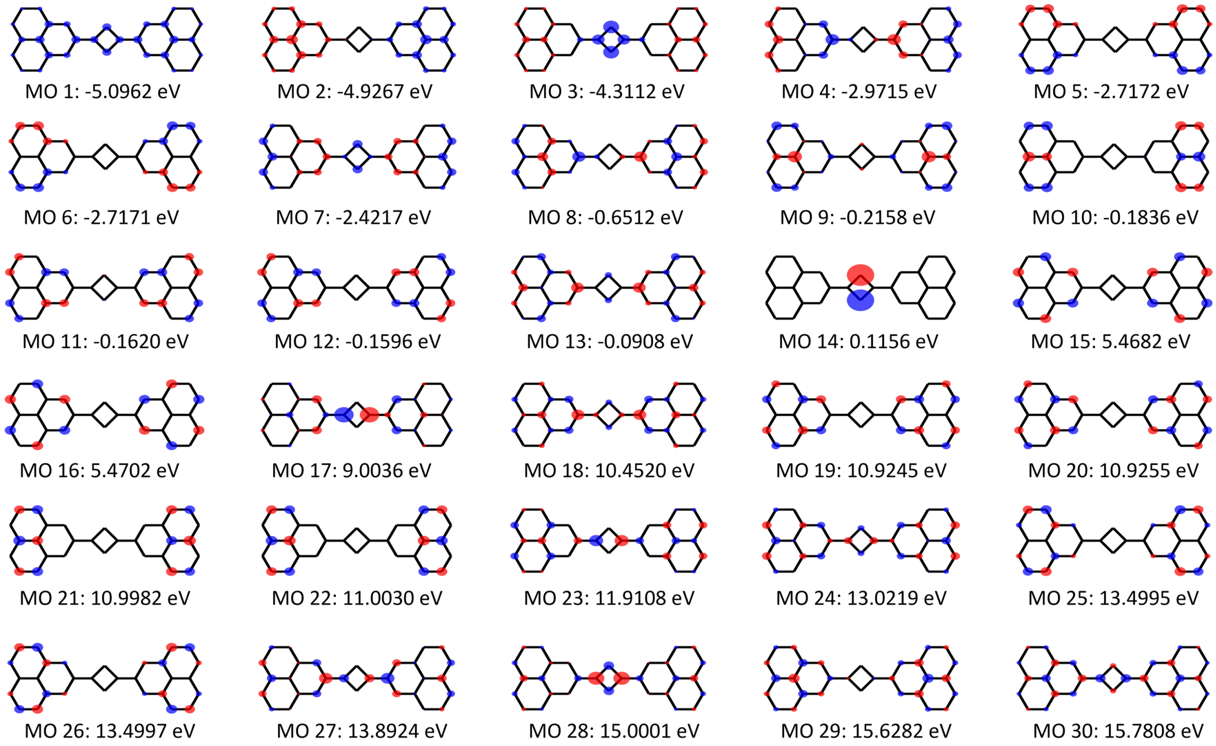

Figure S5: Molecular orbitals obtained at the PPP–Hartree–Fock level for C_2N_2–$(PLY^{\bullet})_2$, shown together with their corresponding orbital energies. Orbital 14 corresponds to the HOMO, orbitals 15 and 16 are the degenerate $SOMO_1$ and $SOMO_2$, and orbital 17 is the LUMO. The PPP model parameters are identical to those used in the main text.

S1.4 The PPP SOMO-LUMO exchange integral

As discussed in the main text, the magnitude of the exchange interaction between the two spin centers in the excited state is governed by the exchange integral between the SOMOs and the bridge LUMO. Within the PPP formalism, this quantity is expressed as:

$$K_{SOMO_1-LUMO} = \frac{e^2}{4\pi\varepsilon_0}\left\langle \psi_{SOMO_1}(1)\psi_{LUMO}(2)\left|\frac{1}{r_{12}}\right|\psi_{LUMO}(1)\psi_{SOMO_1}(2)\right\rangle \tag{S8}$$

By expanding the MOs in the atomic $2p_z$ basis and invoking the zero differential overlap approximation, this expression reads:

$$K_{SOMO_1-LUMO} = \sum_{\mu\nu} c_{SOMO_1,\nu}c_{LUMO,\nu}c_{LUMO,\mu}c_{SOMO_1,\mu}V_{\nu\mu} \tag{S9}$$

Here, the double sum extends over atomic sites μ and ν, $c_{SOMO_1,\mu}$ and $c_{LUMO,\mu}$ denote the AO coefficients of the $SOMO_1$ and LUMO, respectively, and $V_{\nu\mu}$ is the inter-site Coulomb repulsion defined through the Ohno parametrization (cf. Eq. S1). The above expression makes explicit that a finite exchange interaction arises only when the SOMO and LUMO possess overlapping amplitude on the same atomic sites. Consequently, if the two orbitals are spatially disjoint, the exchange integral vanishes.

S2 *Ab initio* Computational Details

S2.1 Computational Setup and Electronic Structure Methods

Molecular structures of the InveST-bridged diradicals shown in Fig. 1c of the main text were optimized at the DFT level using the UBHandHLYP functional[9,10] together with the def2-TZVP basis set in the gas phase. All systems were optimized in their triplet ground states. The calculated vibrational frequencies for the three systems are reported in Tables S20, S21, and S22. No imaginary frequencies were found. To probe conformational flexibility, the potential energy surface was explored by rigidly varying the torsional angle θ associated with rotation about the two bonds connecting the InveST core to the radical fragments, using increments of 5°. For all three diradicals, the minimum of the torsional potential corresponds to a planar geometry with $\theta_{\mathrm{eq}} = 0°$, both for the ground-state (S_0/T_0) and lowest excited-state (S_1/T_1) manifolds. This was confirmed by excited-state geometry optimizations performed with TD-DFT at the CAM-B3LYP/def2-TZVP level for the S_1 and T_1 states (see Section S8). The CAM-B3LYP functional was selected for excited-state calculations due to its range-separated form, which provides a more reliable description of the multiresonant charge-transfer character of the InveST excited states. Ground-state geometry optimizations were carried out using the Gaussian 16 software package,[11] while excited-state optimizations were performed with ORCA (version 5.0.3).[12]

Multireference electronic structure calculations were performed starting from the UBHandHLYP-optimized triplet ground state geometries. CASSCF calculations were first employed to describe static correlation effects, after which dynamic correlation was accounted for by applying the van Vleck quasi-degenerate formulation of strongly contracted second-order N-electron valence perturbation theory (QD-NEVPT2).[13,14] All CASSCF/QD-NEVPT2 calculations were carried out using the ORCA software package (version 5.0.3).[12] Two different active spaces were considered in the CASSCF calculations, namely (4,4) and (6,6), with results obtained using the larger active space discussed in Section S2.2. In all

cases, the def2-TZVP basis set was employed together with the Resolution of Identity (RI) approximation, using the def2/JK auxiliary basis. For each active space, the lowest two singlet and two triplet electronic states were included in the state-averaged treatment. The (4,4) active space comprises four electrons distributed over four frontier MOs, whereas the (6,6) active space extends this description to six electrons in six orbitals. A comparison of the molecular orbitals generated at the CASSCF(4,4)/def2-TZVP and CASSCF(6,6)/def2-TZVP levels indicates only minor differences between the two choices. Across all systems and for both active-space choices, the second-order perturbative corrections remain moderate in magnitude. For C_2N_2–(allyl$^\bullet$)$_2$, the corrections amount to approximately 0.16 eV for the T_1 state and 0.23 eV for S_1. Similar values are obtained for the asymmetric C_2N_2–(allyl$^\bullet$)–(PLY$^\bullet$) diradical, with corrections of about 0.16 eV for T_1 and 0.21 eV for S_1. In the symmetric C_2N_2–(PLY$^\bullet$)$_2$ system, the perturbative contributions are slightly smaller, ranging from roughly 0.11 eV for T_1 to 0.19 eV for S_1.

S2.2 Active Space Dependence: CASSCF Molecular Orbitals and QD-NEVPT2 Energies

We present results obtained using a larger (6,6) active space, extending the (4,4) configuration used in the main text. Specifically, CASSCF molecular orbitals are shown for two representative θ values: 0° and 25°. Upon enlarging the active space, the key frontier orbitals – HOMO, SOMO$_1$, SOMO$_2$, and LUMO – remain largely unchanged for the three diradical systems discussed here, as shown in Figures S6, S7, and S8. The total energies of the singlet and triplet manifolds display consistent trends across all systems, with S_0 and T_0 remaining nearly degenerate and the triplet excited state T_1 systematically stabilized below S_1. For C_2N_2–(allyl$^\bullet$)$_2$, the planar geometry ($\theta = 0°$) shows a moderate increase in the S_1–T_1 energy gap upon enlarging the active space, from approximately 0.19 eV at the QD-NEVPT2(4,4) level to about 0.27 eV with the (6,6) active space. When the molecule is twisted to $\theta = 25°$, the corresponding singlet–triplet splittings obtained with the two active spaces remain very

close. A comparable behavior is observed for the asymmetric C_2N_2–(allyl$^\bullet$)–(PLY$^\bullet$) diradical. At $\theta = 0°$, the S_1–T_1 gap increases from roughly 0.11 eV within the (4,4) active space to about 0.22 eV when the larger (6,6) space is employed, while at $\theta = 25°$ the excited-state splittings show only minor sensitivity to the active-space size. The symmetric C_2N_2–(PLY$^\bullet$)$_2$ system follows the same overall pattern: at the planar geometry, the S_1–T_1 gap grows from approximately 0.09 eV to 0.13 eV upon expanding the active space, whereas at $\theta = 25°$ the singlet–triplet separations obtained with the two active spaces again remain very similar.

Table S3: Total energies in atomic units of the S_0, T_0, T_1, and S_1 states of C_2N_2-(allyl$^\bullet$)$_2$ for planar ($\theta = 0°$) and twisted ($\theta = 25°$) geometries at the QD-NEVPT2 theory level. Results are shown for two different active spaces.

	QD-NEVPT2(4,4)	QD-NEVPT2(6,6)	QD-NEVPT2(4,4)	QD-NEVPT2(6,6)
	**$\theta = 0°$**		**$\theta = 25°$**	
$E(S_0)$	-418.053404	-418.027528	-418.050282	-418.026079
$E(T_0)$	-418.054191	-418.027482	-418.050749	-418.025953
$E(T_1)$	-417.982219	-417.963030	-417.977620	-417.959693
$E(S_1)$	-417.975188	-417.953130	-417.970210	-417.949920

Table S4: Total energies in atomic units of the S_0, T_0, T_1, and S_1 states of C_2N_2-(allyl$^\bullet$)-(PLY$^\bullet$) for planar ($\theta = 0°$) and twisted ($\theta = 25°$) geometries at the QD-NEVPT2 theory level. Results are shown for two different active spaces.

	QD-NEVPT2(4,4)	QD-NEVPT2(6,6)	QD-NEVPT2(4,4)	QD-NEVPT2(6,6)
	**$\theta = 0°$**		**$\theta = 25°$**	
$E(S_0)$	-800.791282	-800.763643	-800.787825	-800.759284
$E(T_0)$	-800.791644	-800.763711	-800.788053	-800.759310
$E(T_1)$	-800.720137	-800.694535	-800.715767	-800.690650
$E(S_1)$	-800.716275	-800.686412	-800.710892	-800.683135

Table S5: Total energies in atomic units of the S_0, T_0, T_1, and S_1 states of C_2N_2-$(PLY^{\bullet})_2$ for planar ($\theta = 0°$) and twisted ($\theta = 25°$) geometries at the QD-NEVPT2 theory level. Results are shown for two different active spaces.

	QD-NEVPT2(4,4)	QD-NEVPT2(6,6)	QD-NEVPT2(4,4)	QD-NEVPT2(6,6)
	$\boldsymbol{\theta = 0°}$		$\boldsymbol{\theta = 25°}$	
$E(S_0)$	-1183.530869	-1183.506907	-1183.526216	-1183.502380
$E(T_0)$	-1183.531007	-1183.506871	-1183.526290	-1183.502352
$E(T_1)$	-1183.460890	-1183.439393	-1183.455173	-1183.434841
$E(S_1)$	-1183.457580	-1183.434669	-1183.451686	-1183.430363

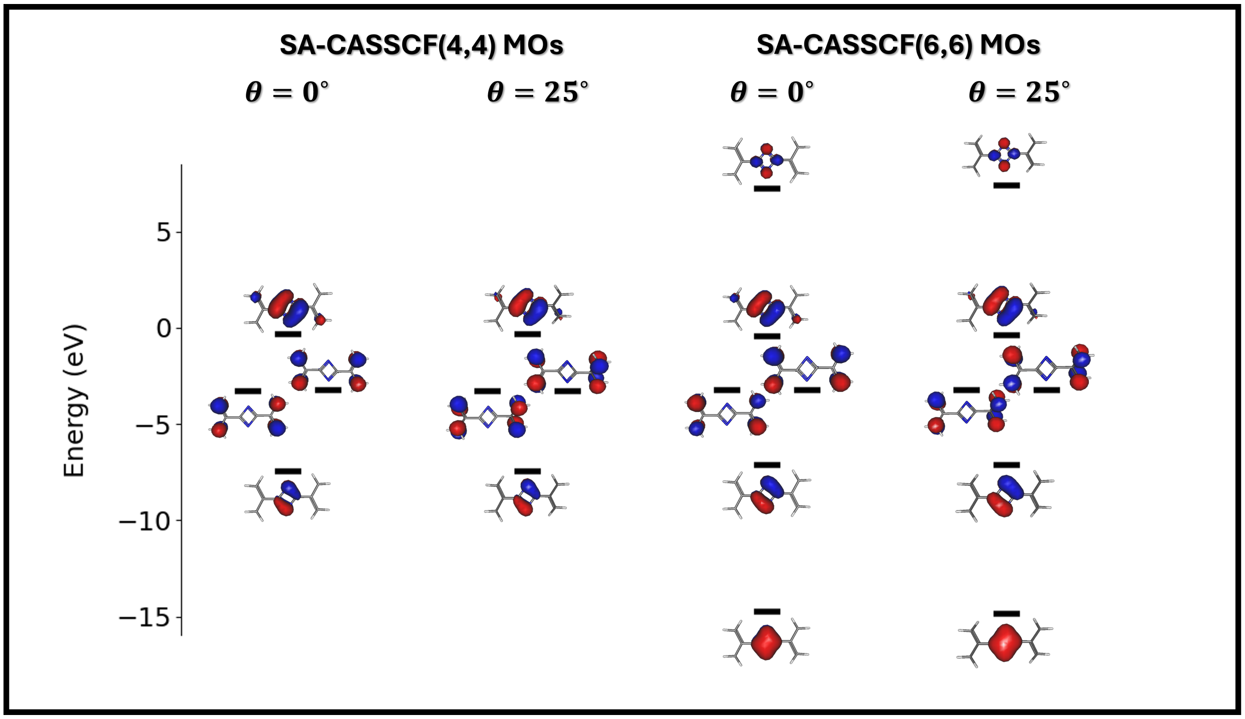

Figure S6: CASSCF frontier molecular orbitals of C_2N_2-$(allyl^{\bullet})_2$ calculated with (4,4) and (6,6) active spaces using the def2-TZVP basis set at $\theta = 0°$ and $\theta = 25°$ torsional angles.

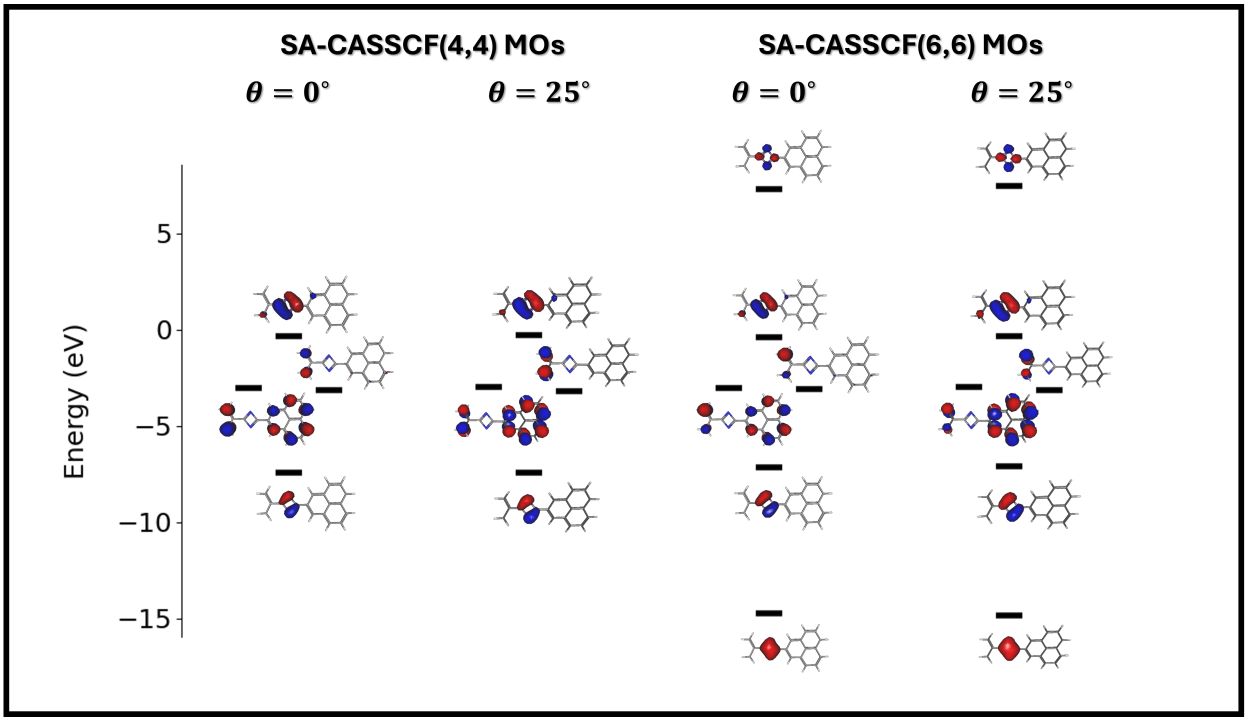

Figure S7: CASSCF frontier molecular orbitals of C_2N_2-(allyl$^\bullet$)-(PLY$^\bullet$) calculated with (4,4) and (6,6) active spaces using the def2-TZVP basis set at $\theta = 0°$ and $\theta = 25°$ torsional angles.

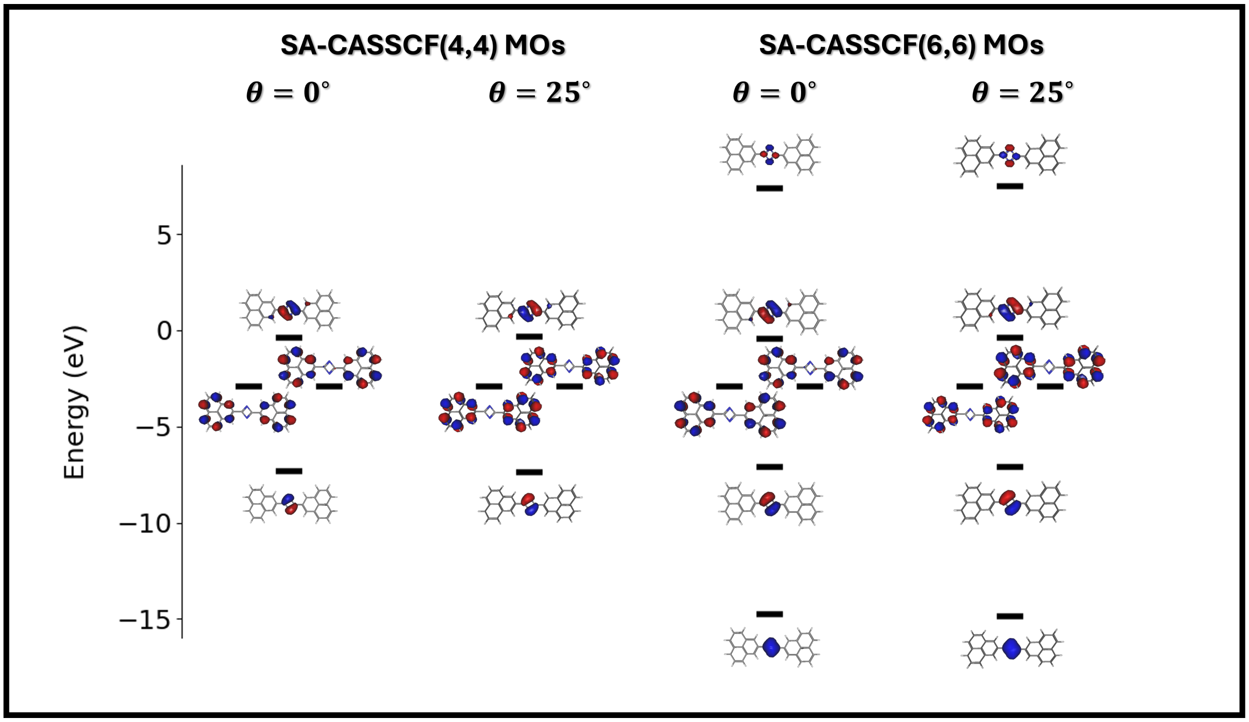

Figure S8: CASSCF frontier molecular orbitals of C_2N_2-$(PLY^{\bullet})_2$ calculated with (4,4) and (6,6) active spaces using the def2-TZVP basis set at $\theta = 0^\circ$ and $\theta = 25^\circ$ torsional angles.

S3 Additional Torsional Scan Results

This section provides complementary information on the torsional behavior of the asymmetric C_2N_2–(allyl$^\bullet$)–(PLY$^\bullet$) (see Fig. S9) and symmetric C_2N_2–(PLY$^\bullet$)$_2$ (see Fig. S10) diradicals. We compare the potential energy surfaces of the lowest singlet and triplet states (S_0, T_0, S_1, and T_1) as functions of the torsional angle θ, computed at both the PPP–RASCI(h,p,hp) and CASSCF(4,4)/QD-NEVPT2 levels. In addition, the frontier MOs relevant to the low-energy electronic structure are shown for the planar geometry ($\theta = 0°$), obtained from CASSCF(4,4) and PPP–HF calculations.

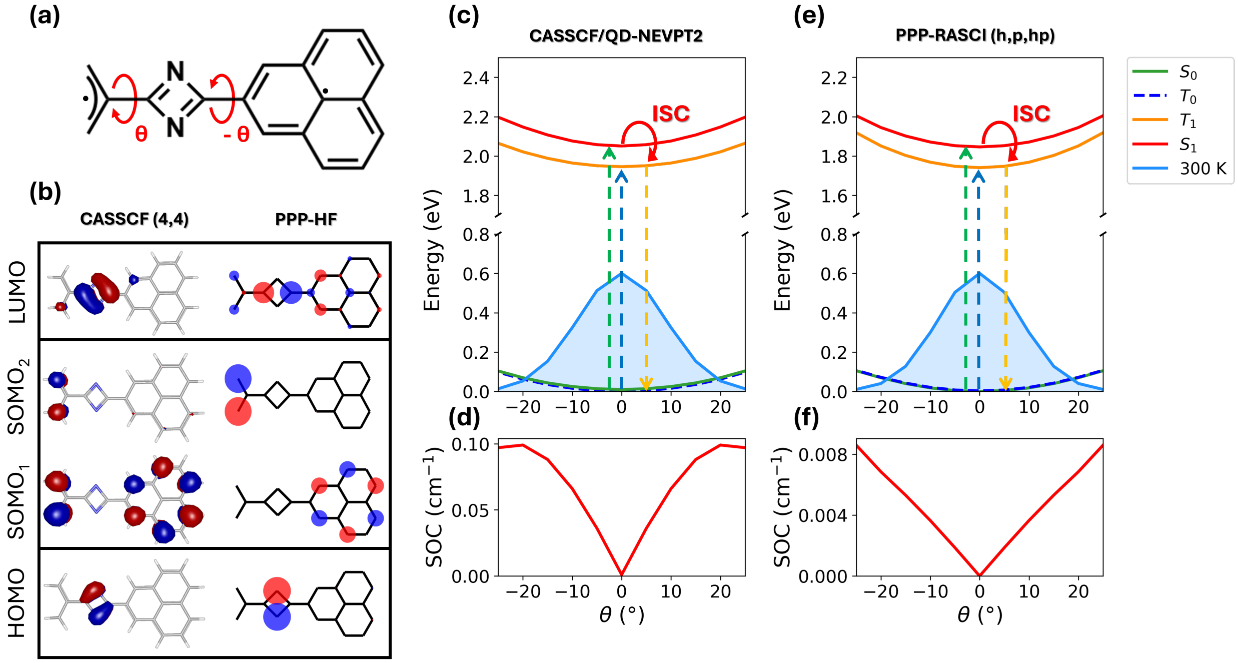

Figure S9: (a) Molecular framework of the asymmetric diradical C_2N_2-(allyl$^\bullet$)-(PLY$^\bullet$), indicating the torsional angle θ. (b) Frontier MOs at the planar geometry ($\theta = 0°$), shown for both PPP–Hartree–Fock and CASSCF(4,4) descriptions. (c) Potential energy profiles of the S_0, T_0, S_1, and T_1 states as functions of θ, calculated at the CASSCF(4,4)/QD-NEVPT2 level, together with the Boltzmann distribution of ground-state θ conformations at room temperature. (d) Torsional dependence of the SOC magnitude between S_1 and T_1, evaluated at the same level of theory. (e,f) Corresponding potential energy curves and SOC trends obtained from PPP–RASCI(h,p,hp) calculations. All PPP model parameters are identical to those defined in the main text.

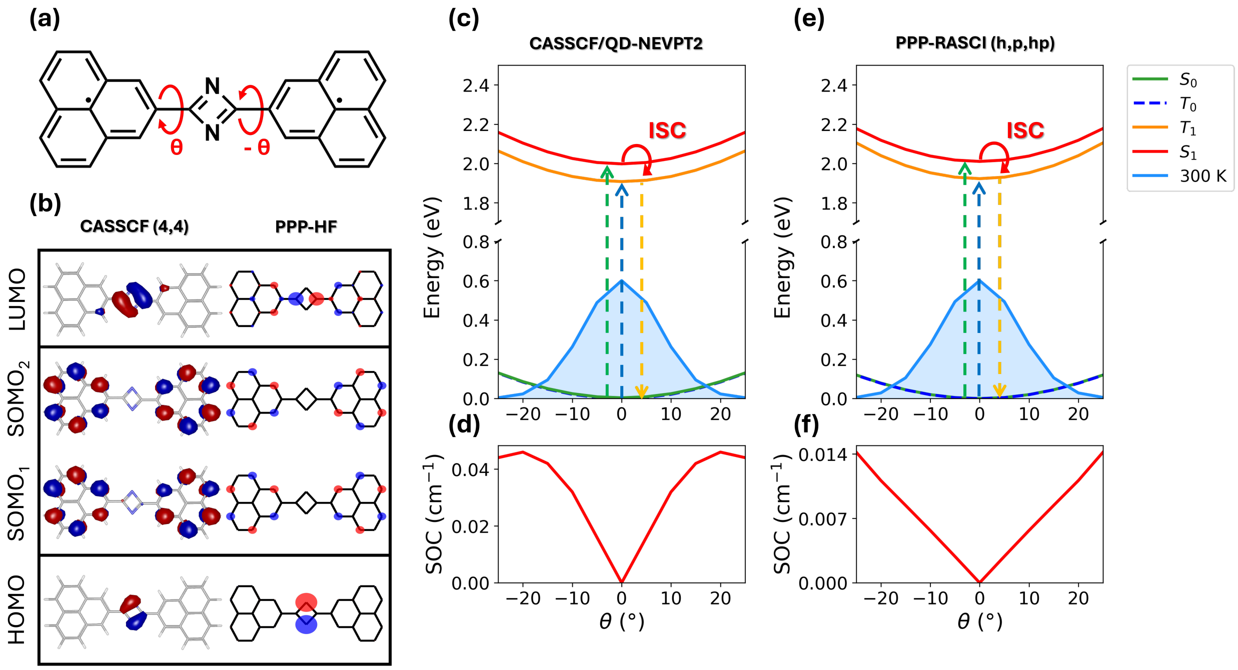

Figure S10: (a) Molecular framework of the symmetric diradical C_2N_2–$(PLY^{\bullet})_2$, highlighting the torsional angle θ defined at the bridge–radical junctions. (b) Frontier molecular orbitals at the planar geometry ($\theta = 0°$), shown for both PPP–Hartree–Fock and CASSCF(4,4) levels of theory. (c) Potential energy surfaces of the S_0, T_0, S_1, and T_1 states as functions of θ, obtained at the CASSCF(4,4)/QD-NEVPT2 level, together with the room-temperature Boltzmann distribution of ground-state torsional conformations. (d) Dependence of the S_1–T_1 SOC magnitude on the torsional angle, evaluated at the same level of theory. (e,f) Corresponding potential energy profiles and SOC trends obtained from PPP–RASCI(h,p,hp) calculations. All PPP parameters are identical to those used in the main text.

S4 Wavefunction Analysis of the S_1 and T_1 Excited States

To clarify the nature of the lowest excited states, we analyzed the CI weights of the S_1 and T_1 wavefunctions for all three C_2N_2-bridged diradicals, using both PPP and CASSCF/QD-NEVPT2 descriptions. The contributions of the dominant electronic configurations were tracked as a function of the torsional angle, with particular attention to the open-shell diradical determinant and to configurations associated with intramolecular SOMO→SOMO charge transfer. As shown in Fig. S11, the electronic structure of both S_1 and T_1 remains largely unchanged over the explored torsional range: in all cases, the wavefunctions are overwhelmingly characterized by the open-shell diradical configuration, whereas the weight of the SOMO→SOMO charge-transfer component remains negligible.

To further quantify this observation, we also analyzed the CI coefficients of the leading configurations in the CASSCF(6,6)/QD-NEVPT2 wavefunctions for the three systems at representative torsional angles ($\theta = 0°$ and $25°$). In all cases, both S_1 and T_1 are largely dominated by the open-shell HOMO→LUMO diradical configuration.

Analysis of the CASSCF(4,4)/QD-NEVPT2 wavefunctions reveals that, for C_2N_2–(allyl$^\bullet$)$_2$, the lowest triplet excited state T_1 is strongly dominated by the HOMO→LUMO diradical configuration, with a weight of 0.93 at the planar geometry ($\theta = 0°$), increasing to 0.97 upon twisting to $\theta = 25°$. The corresponding singlet excited state S_1 exhibits a slightly more mixed character, with the diradical contribution rising from 0.80 at $\theta = 0°$ to 0.92 at $\theta = 25°$. A similar behavior is found for the asymmetric C_2N_2–(allyl$^\bullet$)–(PLY$^\bullet$) system, where the HOMO→LUMO diradical configuration accounts for 0.96 (0.98) of the T_1 wavefunction and 0.85 (0.94) of the S_1 wavefunction at $\theta = 0°$ ($\theta = 25°$). For C_2N_2–(PLY$^\bullet$)$_2$, the diradical character is even more pronounced, with weights of 0.97 and 0.99 in T_1, and 0.92 and 0.97 in S_1, at $\theta = 0°$ and $\theta = 25°$, respectively. Across all three systems and throughout the explored torsional range, the contribution of the SOMO→SOMO charge-transfer configuration remains negligible.

An analogous analysis at the PPP level yields a fully consistent qualitative picture. At the

planar geometry ($\theta = 0°$), the lowest excited states of all three diradicals are again dominated by the HOMO→LUMO diradical configuration. For C_2N_2–(allyl$^\bullet$)$_2$, the corresponding CI squared weights are 0.93 in S_1 and 0.83 in T_1, while values of 0.88 (S_1) and 0.85 (T_1) are obtained for C_2N_2–(allyl$^\bullet$)–(PLY$^\bullet$), and 0.86 (S_1) and 0.80 (T_1) for C_2N_2–(PLY$^\bullet$)$_2$. Unlike the CASSCF(4,4)/QD-NEVPT2 results, where a modest enhancement of the diradical character is observed upon twisting, the PPP CI weights exhibit a weak dependence on θ and remain essentially constant over the explored torsional range.

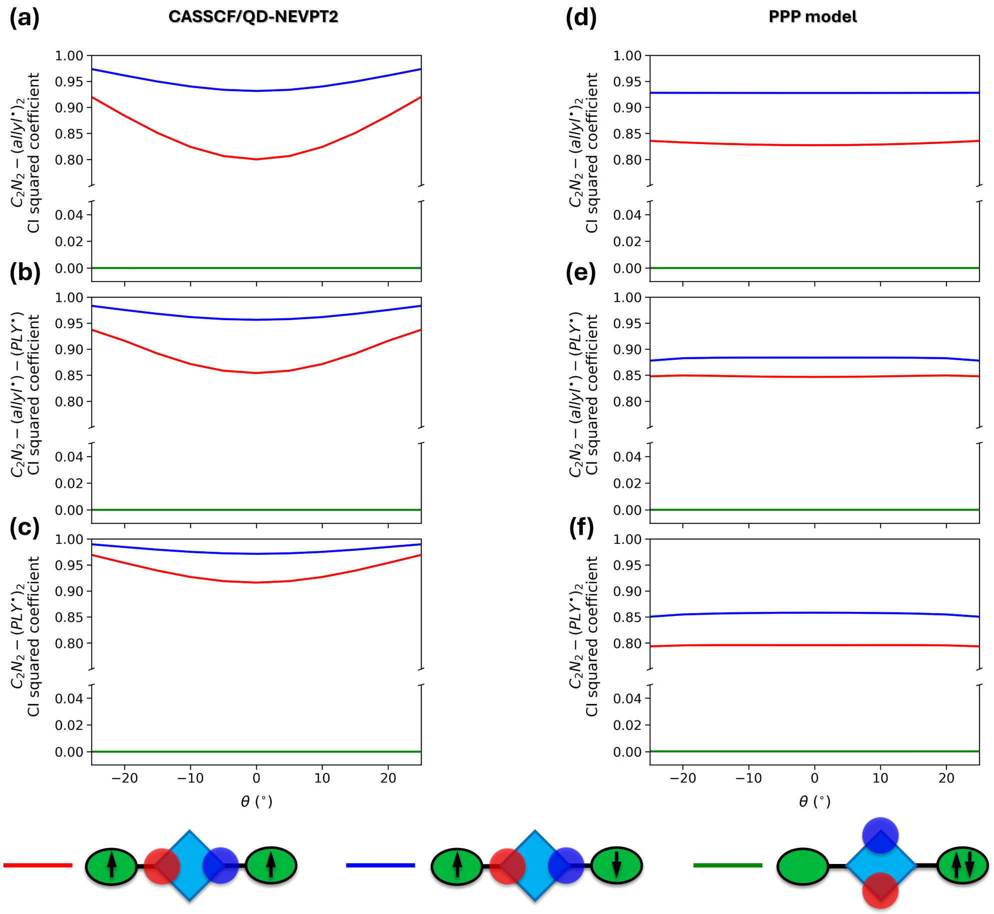

Figure S11: Relative contributions (squared CI coefficients) of selected excited-state configurations as a function of the torsional angle θ. Panels (a–c): CASSCF(4,4)/QD-NEVPT2 results for (a) C_2N_2-(allyl$^\bullet$)$_2$, (b) C_2N_2-(allyl$^\bullet$)-(PLY$^\bullet$), and (c) C_2N_2-(PLY$^\bullet$)$_2$. Panels (d–f): corresponding PPP results. The contributions of the HOMO–LUMO diradical configuration in T_1 (red) and S_1 (blue), as well as the SOMO–SOMO charge-transfer configuration in S_1 (green) are shown. PPP model parameters are the same as in the main text.

S5 Higher-lying excited states

The excited singlet and triplet states obtained from the diagonalization of the PPP Hamiltonian provide direct access to the optical properties of the system. In particular, they allow the evaluation of oscillator strengths associated with spin-allowed transitions originating from the ground states, namely $S_0 \rightarrow S_n$ and $T_0 \rightarrow T_n$ with $n \geq 1$. Within the PPP framework, the electric dipole operator is expressed as:

$$\vec{\mu} = \mu_x \vec{i} + \mu_y \vec{j} = \sum_{\mu} (Z_\mu - n_\mu)(x_\mu \vec{i} + y_\mu \vec{j}) \tag{S10}$$

where x_μ and y_μ are the in-plane Cartesian coordinates of atomic site μ. The oscillator strength corresponding to a transition from the initial state $|g\rangle$ to a final state $|f\rangle$ is then calculated according to:[15]

$$f_{fg} = \frac{2}{3} \frac{m_e}{\hbar e^2} \omega_{fg} |\mu_{fg}|^2 \tag{S11}$$

where $\omega_{fg} = E_f - E_g$ is the transition energy, m_e and e are the electron mass and charge, respectively, and $\mu_{fg} = \langle f|\mu|g\rangle$ is the relevant transition dipole moment.

We report a detailed analysis of the vertical transition energies of the three diradicals at the planar geometry, calculated at the PPP-RASCI(h,p,hp) level. Due to the symmetric nature of the C_2N_2 bridge, the S_1 and T_1 excited states are optically dark, so that photoexcitation necessarily involves higher-lying electronic states. Tables S6, S7, and S8 summarize the low-energy singlet and triplet manifolds, reporting excitation energies, oscillator strengths, dominant electronic configurations, and the corresponding squared CI weights.

For the C_2N_2–(allyl$^\bullet$)$_2$ system, the first bright optical transitions correspond to the $S_0 \rightarrow S_3$ and $T_0 \rightarrow T_5$ excitations at 2.39 eV and 2.30 eV, respectively, both mainly described by a SOMO→LUMO transition. Below these bright states lies a set of optically dark excited states that are central to the relaxation dynamics. These include S_2 and T_2, which

share the same electronic character as S_1 and T_1, as well as T_3, arising from a mixed HOMO/SOMO→SOMO/LUMO double excitation, and T_4, which also involves the radical SOMOs. Together, these dark states provide efficient internal-conversion pathways that funnel population toward S_1 and T_1 excited states.

The same qualitative picture applies to C_2N_2–(allyl$^\bullet$)-(PLY$^\bullet$) and C_2N_2–(PLY$^\bullet$)$_2$: the lowest optically allowed transitions involve excitations from the radical SOMOs to the LUMO of the InveST bridge, followed by rapid internal conversion through intermediate dark states. This relaxation pathway ultimately channels the excited-state population into the lowest singlet and triplet manifolds, S_1 and T_1.

We also examined the possible presence of an inter-SOMO charge-transfer (CT) singlet excitation of the SOMO→SOMO type at the planar geometry. Within the PPP-RASCI(h,p,hp) framework, this CT state is found at 4.256 eV for C_2N_2–(allyl$^\bullet$)$_2$, 4.067 eV for C_2N_2–(allyl$^\bullet$)–(PLY$^\bullet$), and 3.631 eV for C_2N_2–(PLY$^\bullet$)$_2$, i.e., well above the HOMO→LUMO S_1/T_1 pair discussed in the main text. Corresponding *ab initio* QD-NEVPT2 calculations locate the CT singlet state at 3.571 eV, 4.136 eV, and 2.328 eV for the three systems, respectively. The relatively high energy of the CT configuration reflects the absence of strong electron-withdrawing substituents on the radical units capable of stabilizing a zwitterionic state.

Table S6: Transition energies (in eV) for the first three excited singlet states and five excited triplet states of C_2N_2-(allyl$^\bullet$)$_2$ in the planar geometry, calculated at the PPP-RASCI(h,p,hp) level of theory. Oscillator strengths are reported in parentheses. For each excited state, the dominant electronic configuration and the corresponding squared CI amplitude are also given.

	**PPP-RASCI(h,p,hp)**	**Leading Configuration**	**CI weights**
$E(S_1)$	1.751 (0.0000)	HOMO→LUMO	0.928
$E(S_2)$	2.389 (0.0000)	HOMO→LUMO	0.450
$E(S_3)$	2.391 (0.0353)	SOMO→LUMO	0.448
$E(T_1)$	1.605 (0.0000)	HOMO→LUMO	0.827
$E(T_2)$	1.815 (0.0000)	HOMO→LUMO	0.880
$E(T_3)$	1.992 (0.0003)	HOMO/SOMO→SOMO/LUMO	0.848
$E(T_4)$	2.295 (0.0000)	SOMO→LUMO	0.557
$E(T_5)$	2.296 (0.0332)	SOMO→LUMO	0.557

Table S7: Transition energies (in eV) for the first two excited singlet states and three excited triplet states of C_2N_2-(allyl$^{\bullet}$)-(PLY$^{\bullet}$) in the planar geometry, computed at the PPP-RASCI(h,p,hp) level of theory. Oscillator strengths are reported in parentheses. For each excited state, the dominant electronic configuration and the corresponding squared CI amplitude are also given.

	**PPP-RASCI(h,p,hp)**	**Leading Configuration**	**CI weights**
$E(S_1)$	1.846 (0.0000)	HOMO→LUMO	0.884
$E(S_2)$	2.127 (0.0150)	SOMO→LUMO	0.562
$E(T_1)$	1.605 (0.0000)	HOMO→LUMO	0.847
$E(T_2)$	1.815 (0.0000)	HOMO→LUMO	0.796
$E(T_3)$	1.992 (0.0127)	SOMO→LUMO	0.543

Table S8: Transition energies (in eV) for the first two excited singlet states and two excited triplet states of C_2N_2-(PLY$^{\bullet}$)$_2$ in the planar geometry, computed at the PPP-RASCI(h,p,hp) level of theory. Oscillator strengths are reported in parentheses. For each excited state, the dominant electronic configuration and the corresponding squared CI amplitude are also given.

	**PPP-RASCI(h,p,hp)**	**Leading Configuration**	**CI weights**
$E(S_1)$	2.032 (0.0000)	HOMO→LUMO	0.859
$E(S_2)$	2.145 (0.0332)	SOMO→LUMO	0.522
$E(T_1)$	1.942 (0.0000)	HOMO→LUMO	0.796
$E(T_2)$	2.084 (0.0318)	SOMO→LUMO	0.587

S6 Effect of Radical Attachment Topology

To assess the role of the radical-bridge connection site, calculations were performed on a modified system in which the phenalenyl radical units are attached to the InveST bridge through atomic sites bearing finite SOMO amplitude, denoted C_2N_2–$(PLY^{\bullet})'_2$. Following the computational protocol adopted for the three systems discussed in the main text, PPP-RASCI(h,p,hp) calculations were carried out including 4 electrons in 5 MOs. The PPP results were obtained using the idealized geometry described in Section S1.1. At the *ab initio* level, CASSCF(4,4)/QD-NEVPT2 calculations were performed on the DFT-optimized geometry (BHandHLYP/def2-TZVP), reported in Table S19. To improve consistency between the PPP and *ab initio* descriptions for this system, a minor adjustment of the nitrogen PPP parameters was introduced ($\varepsilon_N = -4$ eV, $U_N = 13.5$ eV).

As shown in Fig.S12, attachment through sites with finite SOMO amplitude leads to strong mixing between the radical SOMOs and the InveST bridge orbitals. The two degenerate SOMOs characteristic of the disjoint diradical systems are no longer preserved, indicating a loss of the open-shell diradical character. Indeed, the ground state becomes a closed-shell singlet S_0, while the lowest triplet state T_0 corresponds to a HOMO→LUMO excitation. The first singlet excited state S_1 retains predominantly HOMO→LUMO character, whereas T_1 is mainly described by a HOMO–1→LUMO transition. The $S_0 \rightarrow S_1$ oscillator strength is 0.001 at both the PPP-RASCI and QD-NEVPT2 levels.

Table S9: Absolute energies of the lowest singlet and triplet states (S_0, T_0, S_1, and T_1) of C_2N_2-$(PLY^{\bullet})'_2$ calculated at the CASSCF(4,4)/QD-NEVPT2 level (in atomic units) and at the PPP-RASCI(h,p,hp) level (in eV). The *ab initio* calculations were performed at the DFT-optimized geometry (BHandHLYP/def2-TZVP), whereas the PPP results correspond to the idealized geometry described in Section S1.1.

	**CASSCF(4,4)/QD-NEVPT2 (a.u.)**	**PPP-RASCI(h,p,hp) (eV)**
$E(S_0)$	-1183.527566	-77.325421
$E(T_0)$	-1183.523019	-77.207213
$E(S_1)$	-1183.508932	-76.536455
$E(T_1)$	-1183.500500	-76.823985

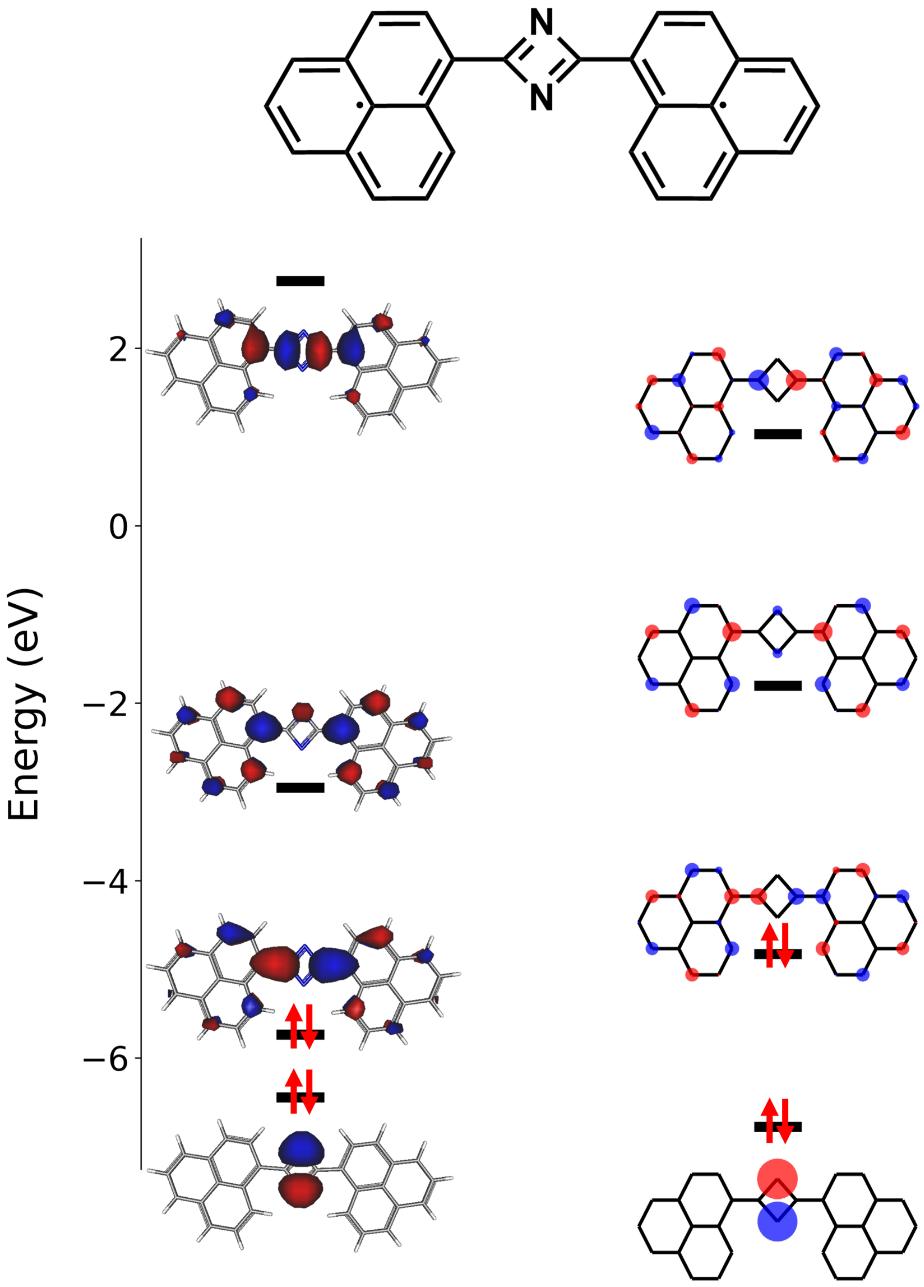

Figure S12: Frontier MOs of C_2N_2–$(PLY^{\bullet})'_2$ obtained at the CASSCF(4,4) level (left column) and at the PPP–Hartree–Fock level (right column). In both cases, the orbitals shown correspond to HOMO-1, HOMO, LUMO, and LUMO+1.

S7 ISC and RISC Rate Calculation Details

S7.1 Construction of the Diabatic Hamiltonian

To construct the diabatic Hamiltonian employed in the ISC and RISC rate calculations (Eq. 3 in the main text), we used a strategy based on reproducing the torsional potential energy landscapes. The parameters entering the model, namely τ_0, β_0, ω_t, a, $2z$, and $2s$, were determined by fitting the eigenvalues of the diabatic Hamiltonian to the adiabatic potential energy curves as functions of the torsional coordinate θ. Separate fitting procedures were carried out for the PPP–RASCI and *ab initio* QD-NEVPT2 datasets, reflecting the differences between the corresponding adiabatic energy profiles. During the fitting, SOC was neglected by setting $V_{SOC} = 0$. Given the relatively small magnitude of SOC in these systems, this approximation does not affect the overall shape of the adiabatic energy surfaces. Under these conditions, the singlet (S_0/S_1) and triplet (T_0/T_1) manifolds can be treated independently. For each spin manifold, the diabatic parameters were optimized such that the resulting eigenvalues closely follow the corresponding adiabatic curves over the full torsional range. Once a satisfactory reproduction of the adiabatic surfaces was achieved, the SOC term was incorporated back into the diabatic Hamiltonian. Within this framework, V_{SOC} is taken as a constant, independent of θ. Its numerical value was selected to ensure that the matrix element $|\langle S_1|V_{SOC}|T_1\rangle|$, calculated using the diabatic eigenstates, reproduces the torsional dependence of the SOC obtained from the underlying electronic-structure calculations, either PPP–RASCI or QD-NEVPT2, depending on the dataset used for the fit.

S7.2 Vibronic Model for Torsional Dynamics

In C_2N_2-bridged diradicals, the small energy gap between the S_1 and T_1 states makes a purely classical description of torsional motion insufficient. Accurate modeling of spin-forbidden transitions therefore requires an explicit quantum mechanical treatment of the torsional degree of freedom. To this end, the diabatic Hamiltonian is constructed in a vibronic basis

formed by the direct product of the four electronic diabatic states and the eigenfunctions of a quantum harmonic oscillator associated with the torsional coordinate θ. The latter is expressed in dimensionless form through bosonic ladder operators as:

$$\theta = (a^\dagger + a)/\sqrt{2} \tag{S12}$$

where $a^\dagger$ and a denote the creation and annihilation operators of the torsional mode, respectively. Because the torsional frequency is very low, a large vibrational basis is required to achieve numerical convergence. Here, 280 torsional eigenstates were used, leading to a total vibronic Hamiltonian dimension of 560 for both the singlet and triplet manifolds. The torsional dependence of the electronic couplings is incorporated by expanding the function $\cos(2\theta)\sin(2\theta)$ in a power series of θ. Consistently with the quartic form adopted for the torsional potential, this expansion is truncated at third order,

$$\cos 2\theta \sin 2\theta \simeq 2\theta - \frac{8}{3}\theta^3 \tag{S13}$$

The resulting vibronic Hamiltonian is first diagonalized with the SOC term set to zero, yielding uncoupled singlet and triplet vibronic eigenstates. These states provide the basis for the subsequent evaluation of ISC and RISC rates, which are computed using Fermi Golden Rule.

Diagonalization of the vibronic Hamiltonian, parametrized to reproduce the adiabatic potential energy surfaces obtained at the QD-NEVPT2 level, yields the vibronic levels of the S_1 and T_1 manifolds. These vibronic states are illustrated in Fig. S13 (panels a, c, and e), where singlet and triplet levels are shown for each of the three diradicals. Using these vibronic eigenstates, ISC and RISC rate constants were subsequently evaluated for a range of relaxation times τ. The resulting rates are summarized in panels b, d, and f.

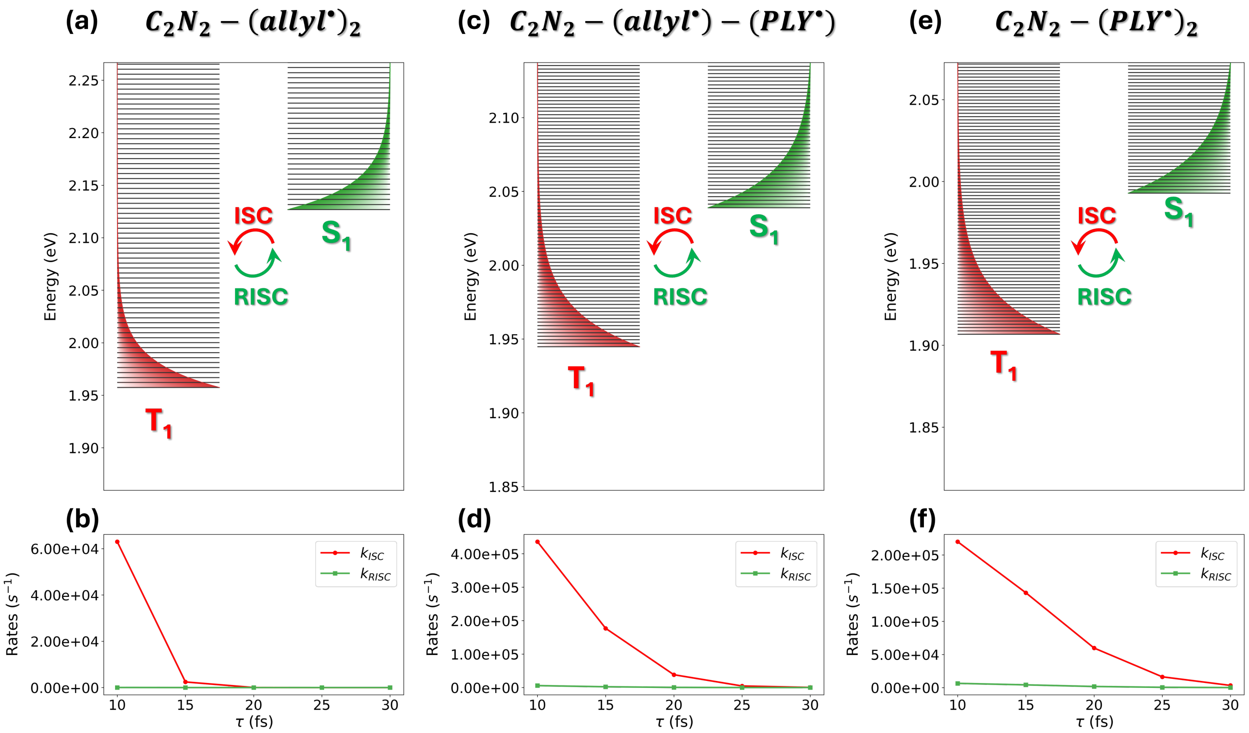

Figure S13: Schematic illustration of the vibronic framework used to calculate ISC and RISC rates for C_2N_2–(allyl$^{\bullet}$)$_2$ (panel a), C_2N_2–(allyl$^{\bullet}$)–(PLY$^{\bullet}$) (panel c), and C_2N_2–(PLY$^{\bullet}$)$_2$ (panel e), based on the QD-NEVPT2 results presented in Fig. 3 of the main text and in Figs. S9 and S10. In each case, the vibronic triplet and singlet eigenstates are shown as black lines. The overall ISC rate is obtained by summing all $S_1 \rightarrow T_1$ transition rates and averaging over the thermally populated singlet states, depicted by the green shaded region. RISC rates are obtained from the ISC rates by enforcing microscopic reversibility. The resulting ISC and RISC rate constants, evaluated for different values of the relaxation time τ, are shown in panels b, d, and f.

S7.3 Temperature dependence of ISC and RISC rates

In Figure S14, the ISC and RISC rates are reported as a function of temperature (100 K, 200 K, and 300 K), calculated following the procedure described in the main text and in Section S7.2. The relaxation time was kept fixed at τ = 10 fs. Results are shown for C_2N_2–(allyl$^{\bullet}$)$_2$, C_2N_2–(allyl$^{\bullet}$)–(PLY$^{\bullet}$), and C_2N_2–(PLY$^{\bullet}$)$_2$, using both PPP and *ab initio* QD-NEVPT2 electronic-structure input data. In all systems and for both levels of electronic structure, a moderate decrease in the ISC rate is observed upon lowering the temperature. However, the rates remain within the same order of magnitude across the 100-300 K interval.

The corresponding RISC rates remain negligible throughout this temperature range.

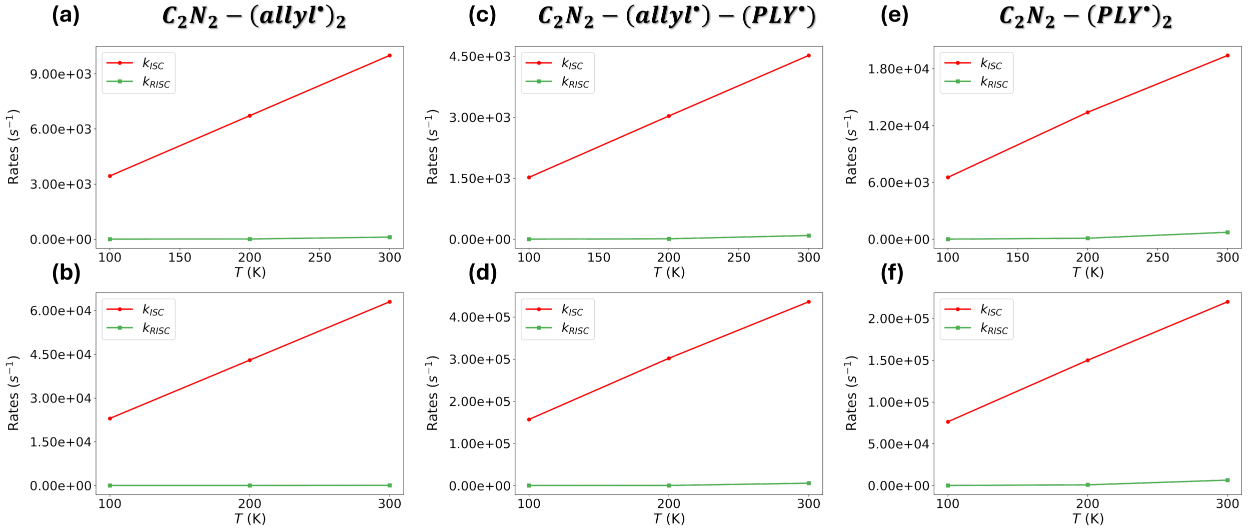

Figure S14: Temperature dependence of the ISC and RISC rates in the 100–300 K range at fixed relaxation time $\tau = 10$ fs. Panels (a,c,e) report results obtained using PPP electronic-structure input data, while panels (b,d,f) show the corresponding rates derived from *ab initio* QD-NEVPT2 input data. Results are shown for C_2N_2–(allyl$^\bullet$)$_2$ (a,b), C_2N_2–(allyl$^\bullet$)–(PLY$^\bullet$) (c,d), and C_2N_2–(PLY$^\bullet$)$_2$ (e,f). RISC rates are obtained from the corresponding ISC rates by enforcing microscopic reversibility.

S8 Cartesian Coordinates and Vibrational Frequencies

Table S10: Cartesian coordinates for C_2N_2-(allyl$^\bullet$)$_2$ optimized geometry in the triplet ground state obtained at DFT level (UBHandHLYP/def2-TZVP).

atom symbol	x (Å)	y (Å)	z (Å)
C	-0.899323	0.058472	0.000008
N	0.075703	-1.047627	0.000036
N	-0.075694	1.047614	0.000012
C	0.899317	-0.058471	0.000007
C	2.340230	0.012079	-0.000009
C	-2.340233	-0.012074	0.000001
C	3.053553	-1.178536	0.000015
H	4.126927	-1.172691	0.000000
C	2.904584	1.268061	-0.000048
H	2.285358	2.143511	-0.000063
C	-2.904583	-1.268060	0.000001
H	-3.971397	-1.391036	-0.000012
C	-3.053555	1.178541	-0.000010
H	-2.541147	2.120725	-0.000009
H	-4.126929	1.172700	-0.000023
H	3.971398	1.391033	-0.000063
H	2.541145	-2.120720	0.000048
H	-2.285350	-2.143503	0.000003

Table S11: Cartesian coordinates for C_2N_2-(allyl$^\bullet$)$_2$ optimized geometry in the first excited triplet state T_1 obtained at TD-DFT level (CAM-B3LYP/def2-TZVP).

atom symbol	x (Å)	y (Å)	z (Å)
C	-0.881395	0.007898	-0.000005
N	0.036924	1.058214	0.000056
N	-0.036857	-1.058218	0.000051
C	0.881498	-0.007899	-0.000006
C	2.313956	-0.052081	-0.000009
C	-2.313837	0.052087	-0.000011
C	2.964605	-1.273914	-0.000030
H	4.044337	-1.326838	-0.000032
C	3.036531	1.133629	0.000011
H	2.518478	2.082046	0.000020
C	-2.964619	1.273782	-0.000034
H	-4.044370	1.326590	-0.000039
C	-3.036551	-1.133492	0.000002
H	-2.518581	-2.081961	0.000009
H	-4.117447	-1.123330	-0.000008
H	4.117416	1.123588	0.000008
H	2.391745	-2.190439	-0.000052
H	-2.391832	2.190364	-0.000052

Table S12: Cartesian coordinates for C_2N_2-(allyl$^{\bullet}$)$_2$ optimized geometry in the first excited singlet state S_1 obtained at TD-DFT level (CAM-B3LYP/def2-TZVP).

atom symbol	x (Å)	y (Å)	z (Å)
C	-0.887406	0.022326	-0.000138
N	0.026456	1.055697	-0.000413
N	-0.026453	-1.055694	-0.000343
C	0.887408	-0.022322	-0.000136
C	2.339801	-0.058142	-0.000009
C	-2.339802	0.058146	-0.000015
C	2.938006	-1.309690	0.001290
H	4.013270	-1.407614	0.001325
C	2.999865	1.161797	-0.001181
H	2.441007	2.084642	-0.002083
C	-2.938006	1.309698	0.001291
H	-4.013276	1.407624	0.001320
C	-2.999866	-1.161799	-0.001196
H	-2.441001	-2.084647	-0.002110
H	-4.078692	-1.205817	-0.001223
H	4.078686	1.205810	-0.001210
H	2.333131	-2.203085	0.002349
H	-2.333128	2.203096	0.002364

Table S13: Cartesian coordinates for C_2N_2-(allyl$^\bullet$)-(PLY$^\bullet$) optimized geometry in the triplet ground state obtained at DFT level (UBHandHLYP/def2-TZVP).

atom symbol	x (Å)	y (Å)	z (Å)
C	-1.949996	-0.121486	-0.000050
N	-2.889870	1.014987	-0.000181
N	-2.803227	-1.086592	-0.000110
C	-3.738817	0.048101	-0.000017
C	-5.183529	0.021729	0.000057
C	-5.858746	1.233770	0.000029
H	-6.931806	1.262430	0.000088
C	-5.784464	-1.216384	0.000152
H	-5.190258	-2.108995	0.000176
H	-6.854470	-1.308281	0.000215
H	-5.315921	2.158770	-0.000046
C	4.319122	1.309501	0.000047
C	3.684117	0.055767	0.000029
C	4.405905	-1.151779	0.000032
C	3.584250	2.475855	0.000043
C	1.523401	1.211214	0.000001
C	2.206238	2.438781	0.000020
C	0.122199	1.140366	-0.000021
H	-0.465400	2.039658	-0.000024
C	-0.518984	-0.087257	-0.000037
C	0.207664	-1.274301	-0.000035
C	1.605101	-1.247457	-0.000012
C	2.375400	-2.425315	-0.000008
C	3.752220	-2.365872	0.000014
H	4.323227	-3.276638	0.000017
H	1.872464	-3.375348	-0.000022
H	4.091027	3.423795	0.000057
H	1.637715	3.351111	0.000017
H	-0.317217	-2.211918	-0.000050
H	5.480488	-1.115842	0.000049
H	5.393707	1.348135	0.000065
C	2.270273	0.007158	0.000006

Table S14: Cartesian coordinates for C_2N_2-(allyl$^\bullet$)-(PLY$^\bullet$) optimized geometry in the first excited triplet state T_1 obtained at TD-DFT level (CAM-B3LYP/def2-TZVP).

atom symbol	x (Å)	y (Å)	z (Å)
C	-1.966155	-0.035685	0.000849
N	-2.869104	1.025842	-0.002874
N	-2.842902	-1.090248	0.004778
C	-3.731898	-0.029294	0.001381
C	-5.174902	-0.044102	0.001904
C	-5.837531	1.175464	-0.001300
H	-6.917622	1.220054	-0.001220
C	-5.812139	-1.276051	0.005364
H	-5.228358	-2.184001	0.007609
H	-6.891099	-1.342673	0.005786
H	-5.272540	2.095298	-0.003997
C	4.352687	1.291845	-0.004765
C	3.685727	0.050566	-0.001266
C	4.391829	-1.169116	0.001757
C	3.641051	2.470809	-0.007771
C	1.544753	1.240751	-0.003984
C	2.252990	2.447556	-0.007471
C	0.143174	1.198837	-0.003622
H	-0.432117	2.116750	-0.006330
C	-0.559335	-0.015647	0.000251
C	0.180974	-1.209063	0.003520
C	1.583199	-1.206535	0.002866
C	2.329443	-2.390236	0.005826
C	3.717803	-2.369573	0.005195
H	4.267872	-3.300080	0.007444
H	1.805885	-3.337754	0.008663
H	4.161545	3.418167	-0.010401
H	1.699688	3.378047	-0.009931
H	-0.364801	-2.144705	0.006575
H	5.474126	-1.150673	0.001349
H	5.435044	1.307474	-0.005059
C	2.282510	0.028274	-0.000800

Table S15: Cartesian coordinates for C_2N_2-(allyl$^{\bullet}$)-(PLY$^{\bullet}$) optimized geometry in the first excited singlet state S_1 obtained at TD-DFT level (CAM-B3LYP/def2-TZVP).

atom symbol	x (Å)	y (Å)	z (Å)
C	-1.948868	-0.018538	0.000028
N	-2.860503	1.021745	-0.000135
N	-2.836558	-1.079306	0.000218
C	-3.743488	-0.039042	0.000044
C	-5.191124	-0.055429	0.000026
C	-5.822183	1.179788	-0.000084
H	-6.899612	1.247455	-0.000112
C	-5.794750	-1.304285	0.000121
H	-5.199001	-2.202749	0.000187
H	-6.870486	-1.395087	0.000126
H	-5.246222	2.091071	-0.000139
C	4.345248	1.293711	0.000001
C	3.682402	0.049622	-0.000005
C	4.375643	-1.177809	-0.000031
C	3.636136	2.477418	0.000028
C	1.541733	1.262821	0.000023
C	2.255298	2.477611	0.000043
C	0.142963	1.224065	0.000034
H	-0.429747	2.141388	0.000063
C	-0.529018	-0.002008	0.000021
C	0.172744	-1.211260	-0.000003
C	1.572008	-1.215747	-0.000018
C	2.315282	-2.412622	-0.000021
C	3.695661	-2.378512	-0.000034
H	4.252297	-3.306438	-0.000047
H	1.788045	-3.357073	-0.000047
H	4.169838	3.418717	0.000036
H	1.704892	3.408743	0.000051
H	-0.377144	-2.142462	-0.000024
H	5.457708	-1.165380	-0.000026
H	5.427295	1.307694	-0.000002
C	2.263309	0.032194	0.000004

Table S16: Cartesian coordinates for C_2N_2-($PLY^{\bullet}$)$_2$ optimized geometry in the triplet ground state obtained at DFT level (UBHandHLYP/def2-TZVP).

atom symbol	x (Å)	y (Å)	z (Å)
C	-0.891324	0.090167	0.000164
N	0.034047	-1.052159	0.000335
N	-0.034040	1.052151	0.000334
C	0.891320	-0.090166	0.000173
C	-7.173712	-1.286661	-0.000190
C	-6.527979	-0.038534	-0.000120
C	-7.239190	1.175172	-0.000120
C	-6.448853	-2.459390	-0.000190
C	-4.377196	-1.212513	-0.000047
C	-5.070715	-2.434245	-0.000121
C	-2.975458	-1.153530	0.000025
H	-2.394763	-2.057303	0.000023
C	-2.324395	0.068187	0.000095
C	-3.040159	1.261126	0.000098
C	-4.437715	1.246632	0.000024
C	-5.197881	2.431240	0.000021
C	-6.575004	2.383682	-0.000050
H	-7.138213	3.299306	-0.000052
H	-4.686634	3.376828	0.000074
H	-6.963864	-3.402898	-0.000246
H	-4.509987	-3.351383	-0.000122
H	-2.506527	2.193806	0.000154
H	-8.314056	1.148554	-0.000174
H	-8.248601	-1.316051	-0.000245
C	-5.113573	-0.002144	-0.000048
C	5.197879	-2.431240	0.000045
C	4.437714	-1.246629	0.000046
C	3.040157	-1.261123	0.000115
C	6.575001	-2.383683	-0.000023
C	5.113573	0.002144	-0.000024
C	6.527979	0.038533	-0.000094
C	7.239188	-1.175173	-0.000092
C	7.173714	1.286661	-0.000165
H	8.248602	1.316050	-0.000218
C	6.448855	2.459389	-0.000167
C	5.070717	2.434245	-0.000099
C	4.377196	1.212514	-0.000026
C	2.975459	1.153532	0.000045
C	2.324394	-0.068185	0.000110
H	2.394766	2.057306	0.000044
H	7.138210	-3.299307	-0.000023

Table S16 – continued

atom symbol	x (Å)	y (Å)	z (Å)
H	8.314054	-1.148557	-0.000145
H	6.963866	3.402897	-0.000222
H	4.509990	3.351384	-0.000101
H	4.686630	-3.376826	0.000098
H	2.506526	-2.193803	0.000170
H	8.343445	-1.152473	0.000665
H	6.987807	3.416171	0.000209
H	4.524594	3.363597	-0.000203
H	4.702678	-3.389242	0.000212
H	2.514762	-2.202790	-0.000183

Table S17: Cartesian coordinates for C_2N_2-($PLY^{\bullet}$)$_2$ optimized geometry in the first excited triplet state T_1 obtained at TD-DFT level (CAM-B3LYP/def2-TZVP).

atom symbol	x (Å)	y (Å)	z (Å)
C	-0.884047	-0.021101	-0.001416
N	0.004478	-1.057428	-0.001159
N	-0.004386	1.057709	-0.002232
C	0.883986	0.021391	-0.001600
C	-7.219938	-1.277553	0.001968
C	-6.539627	-0.042474	0.001313
C	-7.227908	1.188022	0.001435
C	-6.521001	-2.466284	0.001827
C	-4.410249	-1.263607	0.000422
C	-5.135483	-2.466303	0.001059
C	-3.007958	-1.237428	-0.000268
H	-2.443140	-2.160919	-0.000345
C	-2.303384	-0.027897	-0.000877
C	-3.015949	1.175716	-0.000843
C	-4.418528	1.192698	-0.000114
C	-5.151480	2.390645	0.000050
C	-6.536821	2.381556	0.000818
H	-7.078645	3.317652	0.000915
H	-4.615286	3.330900	-0.000411
H	-7.056585	-3.405974	0.002350
H	-4.593195	-3.403046	0.000919
H	-2.456733	2.102632	-0.001356
H	-8.310389	1.183738	0.002032
H	-8.302413	-1.280359	0.002559
C	-5.129750	-0.037871	0.000547
C	5.151300	-2.390611	0.001265
C	4.418464	-1.192646	0.000193
C	3.015866	-1.175554	-0.000284
C	6.536672	-2.381636	0.001774
C	5.129762	0.037873	-0.000370
C	6.539609	0.042385	0.000185
C	7.227809	-1.188157	0.001261
C	7.219993	1.277424	-0.000337
H	8.302470	1.280157	0.000126
C	6.521171	2.466197	-0.001405
C	5.135617	2.466280	-0.002004
C	4.410362	1.263653	-0.001485
C	3.008040	1.237526	-0.001989
C	2.303382	0.028071	-0.001326
H	2.443291	2.161062	-0.002904
H	7.078419	-3.317773	0.002565

Table S17 – continued

atom symbol	x (Å)	y (Å)	z (Å)
H	8.310292	-1.183940	0.001656
H	7.056798	3.405856	-0.001760
H	4.593409	3.403068	-0.002872
H	4.615063	-3.330841	0.001701
H	2.456604	-2.102448	0.000218

Table S18: Cartesian coordinates for C_2N_2-$(PLY^{\bullet})_2$ optimized geometry in the first excited singlet state S_1 obtained at TD-DFT level (CAM-B3LYP/def2-TZVP).

atom symbol	x (Å)	y (Å)	z (Å)
C	-0.908256	-0.005910	-0.000097
N	0.006787	-1.044241	-0.000156
N	-0.006790	1.044221	-0.000137
C	0.908252	0.005889	-0.000111
C	-7.201551	-1.283372	0.000825
C	-6.531408	-0.042663	0.000090
C	-7.217609	1.189227	-0.000584
C	-6.498828	-2.469333	0.001464
C	-4.396864	-1.270539	0.000671
C	-5.117065	-2.479271	0.001393
C	-2.995662	-1.239709	0.000576
H	-2.431671	-2.161682	0.001051
C	-2.322213	-0.015119	-0.000067
C	-3.011666	1.200553	-0.000663
C	-4.413165	1.213127	-0.000657
C	-5.149028	2.412373	-0.001340
C	-6.530572	2.384324	-0.001293
H	-7.082045	3.315587	-0.001815
H	-4.617699	3.354463	-0.001883
H	-7.037954	-3.407804	0.002027
H	-4.573459	-3.414335	0.001885
H	-2.459719	2.129790	-0.001165
H	-8.299682	1.182717	-0.000546
H	-8.283602	-1.290941	0.000882
C	-5.111040	-0.033353	0.000031
C	5.149053	-2.412352	-0.001300
C	4.413176	-1.213115	-0.000625
C	3.011681	-1.200558	-0.000653
C	6.530599	-2.384290	-0.001249
C	5.111042	0.033368	0.000061
C	6.531408	0.042698	0.000112
C	7.217623	-1.189186	-0.000550
C	7.201535	1.283415	0.000824
H	8.283585	1.290998	0.000859
C	6.498793	2.469366	0.001473
C	5.117030	2.479282	0.001426
C	4.396846	1.270540	0.000699
C	2.995652	1.239700	0.000617
C	2.322211	0.015105	-0.000047
H	2.431662	2.161674	0.001112
H	7.082079	-3.315549	-0.001759

Table S18 – continued

atom symbol	x (Å)	y (Å)	z (Å)
H	8.299695	-1.182662	-0.000508
H	7.037904	3.407846	0.002023
H	4.573412	3.414338	0.001934
H	4.617735	-3.354448	-0.001843
H	2.459750	-2.129806	-0.001187

Table S19: Cartesian coordinates for C_2N_2-$(PLY^{\bullet})'_2$ optimized geometry in the singlet ground state obtained at DFT level (BHandHLYP/def2-TZVP).

atom symbol	x (Å)	y (Å)	z (Å)
C	-0.8879240	-0.7256810	-0.0002040
N	0.0000080	0.3228780	-0.0001760
N	-0.0000030	-1.7734420	-0.0002300
C	0.8879530	-0.7257820	-0.0001990
C	-2.2795470	-0.8127280	-0.0001630
C	-2.8404750	-2.1159520	-0.0003690
C	-4.1731180	-2.3070190	-0.0003850
C	-5.0832330	-1.2058510	-0.0001620
C	-4.5579140	0.1071920	-0.0000540
C	-3.1579710	0.3260430	-0.0000460
C	-2.6836120	1.6303470	0.0000380
C	-3.5620970	2.7070560	0.0003560
C	-4.9137810	2.5058870	0.0004610
C	-5.4432140	1.2048330	0.0002750
C	-6.4514960	-1.3899260	0.0000550
C	-7.3225030	-0.3042570	0.0002520
C	-6.8304860	0.9693450	0.0003660
C	2.2795680	-0.8128080	-0.0001760
C	2.8405450	-2.1160370	0.0000790
C	4.1732000	-2.3070400	0.0003110
C	3.1579670	0.3259950	-0.0002710
C	4.5579060	0.1071990	0.0000090
C	5.0832770	-1.2058210	0.0003090
C	2.6835770	1.6302700	-0.0005720
C	3.5620180	2.7070150	-0.0005170
C	4.9136820	2.5059030	-0.0002360
C	5.4431610	1.2048650	-0.0000360
C	6.8304520	0.9694280	0.0001460
C	7.3225170	-0.3041590	0.0004920
C	6.4515420	-1.3898550	0.0005690
H	-2.1550420	-2.9417470	-0.0004470
H	-4.5776690	-3.3036790	-0.0004950
H	-6.8453330	-2.3908060	0.0000820
H	-8.3837180	-0.4729450	0.0002380
H	-7.4998470	1.8111080	0.0004260
H	-5.5896590	3.3427410	0.0005820
H	-3.1678440	3.7067440	0.0004290
H	-1.6220250	1.7901270	-0.0000840
H	2.1551440	-2.9418600	0.0000910
H	4.5777980	-3.3036850	0.0004860
H	6.8454100	-2.3907210	0.0008020

Table S19 – continued

atom symbol	x (Å)	y (Å)	z (Å)
H	8.3837380	-0.4728140	0.0006330
H	7.4997890	1.8112120	0.0000140
H	5.5895340	3.3427810	-0.0002530
H	3.1677310	3.7066910	-0.0007950
H	1.6219850	1.7900180	-0.0008370

Table S20: Vibrational frequencies for C_2N_2-(allyl$^\bullet$)$_2$ optimized geometry obtained at DFT level (UBHandHLYP/def2-TZVP).

	wavenumber (cm^{-1})
$\bar{\nu}_1$	65.92
$\bar{\nu}_2$	82.26
$\bar{\nu}_3$	122.49
$\bar{\nu}_4$	135.94
$\bar{\nu}_5$	237.64
$\bar{\nu}_6$	295.43
$\bar{\nu}_7$	323.93
$\bar{\nu}_8$	399.43
$\bar{\nu}_9$	455.20
$\bar{\nu}_{10}$	478.38
$\bar{\nu}_{11}$	530.78
$\bar{\nu}_{12}$	532.38
$\bar{\nu}_{13}$	565.48
$\bar{\nu}_{14}$	585.37
$\bar{\nu}_{15}$	591.21
$\bar{\nu}_{16}$	624.20
$\bar{\nu}_{17}$	727.43
$\bar{\nu}_{18}$	738.88
$\bar{\nu}_{19}$	796.04
$\bar{\nu}_{20}$	811.90
$\bar{\nu}_{21}$	820.41
$\bar{\nu}_{22}$	877.36
$\bar{\nu}_{23}$	877.58
$\bar{\nu}_{24}$	945.94
$\bar{\nu}_{25}$	1011.27
$\bar{\nu}_{26}$	1018.82
$\bar{\nu}_{27}$	1054.96
$\bar{\nu}_{28}$	1058.30
$\bar{\nu}_{29}$	1112.08
$\bar{\nu}_{30}$	1195.48
$\bar{\nu}_{31}$	1377.04
$\bar{\nu}_{32}$	1384.44
$\bar{\nu}_{33}$	1427.34
$\bar{\nu}_{34}$	1431.61
$\bar{\nu}_{35}$	1534.07
$\bar{\nu}_{36}$	1537.39
$\bar{\nu}_{37}$	1562.24
$\bar{\nu}_{38}$	1571.41
$\bar{\nu}_{39}$	1575.62
$\bar{\nu}_{40}$	1628.79
$\bar{\nu}_{41}$	3246.71

Table S20 – continued

	wavenumber (cm^{-1})
$\bar{\nu}_{42}$	3246.78
$\bar{\nu}_{43}$	3252.79
$\bar{\nu}_{44}$	3252.93
$\bar{\nu}_{45}$	3354.78
$\bar{\nu}_{46}$	3354.80
$\bar{\nu}_{47}$	3355.75
$\bar{\nu}_{48}$	3355.79

Table S21: Vibrational frequencies for C_2N_2-(allyl$^\bullet$)-(PLY$^\bullet$) optimized geometry obtained at DFT level (UBHandHLYP/def2-TZVP).

	wavenumber (cm^{-1})
$\bar{\nu}_1$	38.28
$\bar{\nu}_2$	45.83
$\bar{\nu}_3$	61.60
$\bar{\nu}_4$	110.26
$\bar{\nu}_5$	124.81
$\bar{\nu}_6$	180.12
$\bar{\nu}_7$	181.05
$\bar{\nu}_8$	185.66
$\bar{\nu}_9$	222.65
$\bar{\nu}_{10}$	265.22
$\bar{\nu}_{11}$	304.25
$\bar{\nu}_{12}$	357.44
$\bar{\nu}_{13}$	394.30
$\bar{\nu}_{14}$	399.46
$\bar{\nu}_{15}$	447.42
$\bar{\nu}_{16}$	484.84
$\bar{\nu}_{17}$	506.82
$\bar{\nu}_{18}$	508.91
$\bar{\nu}_{19}$	509.79
$\bar{\nu}_{20}$	531.69
$\bar{\nu}_{21}$	542.25
$\bar{\nu}_{22}$	552.34
$\bar{\nu}_{23}$	591.42
$\bar{\nu}_{24}$	591.69
$\bar{\nu}_{25}$	624.80
$\bar{\nu}_{26}$	661.78
$\bar{\nu}_{27}$	662.13
$\bar{\nu}_{28}$	668.19
$\bar{\nu}_{29}$	717.73
$\bar{\nu}_{30}$	728.78
$\bar{\nu}_{31}$	787.36
$\bar{\nu}_{32}$	798.64
$\bar{\nu}_{33}$	805.77
$\bar{\nu}_{34}$	823.04
$\bar{\nu}_{35}$	833.38
$\bar{\nu}_{36}$	879.93
$\bar{\nu}_{37}$	880.66
$\bar{\nu}_{38}$	883.76
$\bar{\nu}_{39}$	893.67
$\bar{\nu}_{40}$	921.12
$\bar{\nu}_{41}$	925.33

Table S21 – continued

	wavenumber (cm^{-1})
$\bar{\nu}_{42}$	937.48
$\bar{\nu}_{43}$	954.17
$\bar{\nu}_{44}$	987.44
$\bar{\nu}_{45}$	1003.53
$\bar{\nu}_{46}$	1008.44
$\bar{\nu}_{47}$	1016.57
$\bar{\nu}_{48}$	1057.69
$\bar{\nu}_{49}$	1078.94
$\bar{\nu}_{50}$	1095.05
$\bar{\nu}_{51}$	1124.30
$\bar{\nu}_{52}$	1147.70
$\bar{\nu}_{53}$	1154.64
$\bar{\nu}_{54}$	1169.40
$\bar{\nu}_{55}$	1217.04
$\bar{\nu}_{56}$	1243.36
$\bar{\nu}_{57}$	1246.09
$\bar{\nu}_{58}$	1257.24
$\bar{\nu}_{59}$	1286.74
$\bar{\nu}_{60}$	1336.57
$\bar{\nu}_{61}$	1378.88
$\bar{\nu}_{62}$	1382.82
$\bar{\nu}_{63}$	1388.15
$\bar{\nu}_{64}$	1424.28
$\bar{\nu}_{65}$	1438.13
$\bar{\nu}_{66}$	1457.67
$\bar{\nu}_{67}$	1479.16
$\bar{\nu}_{68}$	1511.16
$\bar{\nu}_{69}$	1534.63
$\bar{\nu}_{70}$	1540.21
$\bar{\nu}_{71}$	1572.20
$\bar{\nu}_{72}$	1602.20
$\bar{\nu}_{73}$	1613.35
$\bar{\nu}_{74}$	1619.41
$\bar{\nu}_{75}$	1635.09
$\bar{\nu}_{76}$	1649.84
$\bar{\nu}_{77}$	1651.91
$\bar{\nu}_{78}$	1656.97
$\bar{\nu}_{79}$	3246.19
$\bar{\nu}_{80}$	3252.45
$\bar{\nu}_{81}$	3262.37
$\bar{\nu}_{82}$	3264.19
$\bar{\nu}_{83}$	3273.03
$\bar{\nu}_{84}$	3274.72

Table S21 – continued

	wavenumber (cm^{-1})
$\bar{\nu}_{85}$	3286.09
$\bar{\nu}_{86}$	3287.22
$\bar{\nu}_{87}$	3288.61
$\bar{\nu}_{88}$	3293.52
$\bar{\nu}_{89}$	3354.55
$\bar{\nu}_{90}$	3355.61

Table S22: Vibrational frequencies for C_2N_2-$(PLY^{\bullet})_2$ optimized geometry obtained at DFT level (UBHandHLYP/def2-TZVP).

	wavenumber (cm^{-1})
$\bar{\nu}_1$	18.62
$\bar{\nu}_2$	20.73
$\bar{\nu}_3$	30.92
$\bar{\nu}_4$	67.61
$\bar{\nu}_5$	104.20
$\bar{\nu}_6$	113.92
$\bar{\nu}_7$	123.52
$\bar{\nu}_8$	152.64
$\bar{\nu}_9$	175.94
$\bar{\nu}_{10}$	180.31
$\bar{\nu}_{11}$	183.04
$\bar{\nu}_{12}$	191.74
$\bar{\nu}_{13}$	253.38
$\bar{\nu}_{14}$	296.86
$\bar{\nu}_{15}$	299.35
$\bar{\nu}_{16}$	304.78
$\bar{\nu}_{17}$	311.15
$\bar{\nu}_{18}$	378.20
$\bar{\nu}_{19}$	412.81
$\bar{\nu}_{20}$	446.31
$\bar{\nu}_{21}$	449.04
$\bar{\nu}_{22}$	491.63
$\bar{\nu}_{23}$	495.69
$\bar{\nu}_{24}$	499.62
$\bar{\nu}_{25}$	507.34
$\bar{\nu}_{26}$	509.39
$\bar{\nu}_{27}$	509.83
$\bar{\nu}_{28}$	514.15
$\bar{\nu}_{29}$	528.75
$\bar{\nu}_{30}$	536.96
$\bar{\nu}_{31}$	569.73
$\bar{\nu}_{32}$	571.73
$\bar{\nu}_{33}$	592.88
$\bar{\nu}_{34}$	618.61
$\bar{\nu}_{35}$	630.73
$\bar{\nu}_{36}$	657.05
$\bar{\nu}_{37}$	667.13
$\bar{\nu}_{38}$	668.23
$\bar{\nu}_{39}$	668.98
$\bar{\nu}_{40}$	685.09
$\bar{\nu}_{41}$	710.00

Table S22 – continued

	wavenumber (cm^{-1})
$\bar{\nu}_{42}$	730.59
$\bar{\nu}_{43}$	787.03
$\bar{\nu}_{44}$	787.39
$\bar{\nu}_{45}$	797.92
$\bar{\nu}_{46}$	802.11
$\bar{\nu}_{47}$	816.38
$\bar{\nu}_{48}$	831.52
$\bar{\nu}_{49}$	834.43
$\bar{\nu}_{50}$	879.66
$\bar{\nu}_{51}$	881.80
$\bar{\nu}_{52}$	881.92
$\bar{\nu}_{53}$	885.16
$\bar{\nu}_{54}$	893.77
$\bar{\nu}_{55}$	893.96
$\bar{\nu}_{56}$	910.33
$\bar{\nu}_{57}$	925.34
$\bar{\nu}_{58}$	925.50
$\bar{\nu}_{59}$	933.08
$\bar{\nu}_{60}$	938.70
$\bar{\nu}_{61}$	939.09
$\bar{\nu}_{62}$	956.16
$\bar{\nu}_{63}$	956.39
$\bar{\nu}_{64}$	1003.08
$\bar{\nu}_{65}$	1003.09
$\bar{\nu}_{66}$	1008.01
$\bar{\nu}_{67}$	1008.01
$\bar{\nu}_{68}$	1014.07
$\bar{\nu}_{69}$	1077.77
$\bar{\nu}_{70}$	1082.25
$\bar{\nu}_{71}$	1095.08
$\bar{\nu}_{72}$	1095.21
$\bar{\nu}_{73}$	1127.71
$\bar{\nu}_{74}$	1135.28
$\bar{\nu}_{75}$	1151.44
$\bar{\nu}_{76}$	1152.00
$\bar{\nu}_{77}$	1169.16
$\bar{\nu}_{78}$	1169.22
$\bar{\nu}_{79}$	1216.41
$\bar{\nu}_{80}$	1216.97
$\bar{\nu}_{81}$	1231.99
$\bar{\nu}_{82}$	1240.63
$\bar{\nu}_{83}$	1244.18
$\bar{\nu}_{84}$	1245.04

Table S22 – continued

	wavenumber (cm^{-1})
$\bar{\nu}_{85}$	1256.72
$\bar{\nu}_{86}$	1257.14
$\bar{\nu}_{87}$	1286.02
$\bar{\nu}_{88}$	1286.19
$\bar{\nu}_{89}$	1326.58
$\bar{\nu}_{90}$	1336.99
$\bar{\nu}_{91}$	1382.24
$\bar{\nu}_{92}$	1382.35
$\bar{\nu}_{93}$	1387.34
$\bar{\nu}_{94}$	1387.48
$\bar{\nu}_{95}$	1435.45
$\bar{\nu}_{96}$	1438.28
$\bar{\nu}_{97}$	1456.84
$\bar{\nu}_{98}$	1458.23
$\bar{\nu}_{99}$	1479.16
$\bar{\nu}_{100}$	1479.20
$\bar{\nu}_{101}$	1509.86
$\bar{\nu}_{102}$	1512.65
$\bar{\nu}_{103}$	1539.56
$\bar{\nu}_{104}$	1540.99
$\bar{\nu}_{105}$	1610.30
$\bar{\nu}_{106}$	1610.87
$\bar{\nu}_{107}$	1617.06
$\bar{\nu}_{108}$	1619.12
$\bar{\nu}_{109}$	1632.01
$\bar{\nu}_{110}$	1634.84
$\bar{\nu}_{111}$	1646.10
$\bar{\nu}_{112}$	1649.45
$\bar{\nu}_{113}$	1650.90
$\bar{\nu}_{114}$	1652.34
$\bar{\nu}_{115}$	1659.32
$\bar{\nu}_{116}$	1670.23
$\bar{\nu}_{117}$	3262.14
$\bar{\nu}_{118}$	3262.14
$\bar{\nu}_{119}$	3263.97
$\bar{\nu}_{120}$	3263.98
$\bar{\nu}_{121}$	3272.91
$\bar{\nu}_{122}$	3272.91
$\bar{\nu}_{123}$	3274.60
$\bar{\nu}_{124}$	3274.60
$\bar{\nu}_{125}$	3285.98
$\bar{\nu}_{126}$	3285.99
$\bar{\nu}_{127}$	3287.10

Table S22 – continued

	wavenumber (cm^{-1})
$\bar{\nu}_{128}$	3287.12
$\bar{\nu}_{129}$	3288.59
$\bar{\nu}_{130}$	3288.63
$\bar{\nu}_{131}$	3293.69
$\bar{\nu}_{132}$	3293.73

References

(1) Ohno, K. Some remarks on the Pariser-Parr-Pople method. *Theoretica chimica acta* **1964**, *2*, 219–227.

(2) Albert, I. D. L.; Ramasesha, S.; Das, P. K. Properties of some low-lying electronic states in polymethineimines and poly(2,3-diazabutadienes). *Physical Review B* **1991**, *43*, 7013–7019.

(3) Thomas, S.; Pati, Y.; Ramasesha, S. Linear and nonlinear optical properties of expanded porphyrins: A DMRG study. *The Journal of Physical Chemistry A* **2013**, *117*, 7804–7809.

(4) Lehoucq, R.; Sorensen, D.; Yang, C. *ARPACK Users' Guide*; Society for Industrial and Applied Mathematics, 1998.

(5) Bedogni, M.; Di Maiolo, F. Singlet–Triplet Inversion in Triangular Boron Carbon Nitrides. *Journal of Chemical Theory and Computation* **2024**, *20*, 8634–8643.

(6) Casanova, D.; Head-Gordon, M. Restricted active space spin-flip configuration interaction approach: theory, implementation and examples. *Physical Chemistry Chemical Physics* **2009**, *11*, 9779.

(7) Sandoval-Salinas, M. E.; Carreras, A.; Casanova, D. Triangular graphene nanofragments: open-shell character and doping. *Physical Chemistry Chemical Physics* **2019**, *21*, 9069–9076.

(8) Casanova, D. Restricted active space configuration interaction methods for strong correlation: Recent developments. *WIREs Computational Molecular Science* **2022**, *12*, e1561.

(9) Nakano, M.; Champagne, B. Theoretical Design of Open-Shell Singlet Molecular Sys-

tems for Nonlinear Optics. *The Journal of Physical Chemistry Letters* **2015**, *6*, 3236–3256.

(10) Nakano, M.; Champagne, B. Nonlinear optical properties in open-shell molecular systems. *WIREs Computational Molecular Science* **2016**, *6*, 198–210.

(11) Frisch, M. J. et al. Gaussian~16 Revision B.01. 2016; Gaussian Inc. Wallingford CT.

(12) Neese, F. The ORCA program system. *WIRES Comput. Molec. Sci.* **2012**, *2*, 73–78.

(13) Franz, M.; Neese, F.; Richert, S. Calculation of exchange couplings in the electronically excited state of molecular three-spin systems. *Chemical Science* **2022**, *13*, 12358–12366.

(14) Poh, Y. R.; Morozov, D.; Kazmierczak, N. P.; Hadt, R. G.; Groenhof, G.; Yuen-Zhou, J. Alternant Hydrocarbon Diradicals as Optically Addressable Molecular Qubits. *Journal of the American Chemical Society* **2024**, *146*, 15549–15561.

(15) Lakowicz, J. R. *Principles of fluorescence spectroscopy*, 3rd ed.; Springer: New York, 2006.
